# A Novel Vitronectin Peptide Facilitates Differentiation of Oligodendrocytes from Human Pluripotent Stem Cells (Synthetic ECM for Oligodendrocyte Differentiation)

**DOI:** 10.3390/biology10121254

**Published:** 2021-12-01

**Authors:** Won Ung Park, Gyu-Bum Yeon, Myeong-Sang Yu, Hui-Gwan Goo, Su-Hee Hwang, Dokyun Na, Dae-Sung Kim

**Affiliations:** 1 Department of Biotechnology, Korea University, 145 Anam-ro, Seongbuk-gu, Seoul 02841, Korea; wonungpark@naver.com (W.U.P.); dagurim@gmail.com (G.-B.Y.); 2 Institute of Animal Molecular Biotechnology, Korea University, 145 Anam-ro, Seongbuk-gu, Seoul 02841, Korea; 3 Department of Biomedical Engineering, Chung-Ang University, 84 Heukseok-ro, Dongjak-gu, Seoul 06974, Korea; msirang@naver.com (M.-S.Y.); hsh3024@naver.com (S.-H.H.); 4 AMO Life Sciences, 91 Gimpo-daero 1950 beon-gil, Tongjin-eup, Gyeonggi-do 10014, Korea; pensa@amogreentech.co.kr; 5 Department of Pediatrics, Korea University College of Medicine, Guro Hospital, 97 Gurodong-gil, Guro-gu, Seoul 08308, Korea

**Keywords:** oligodendrocytes, differentiation, hPSCs, vitronectin, transcriptomic analysis

## Abstract

**Simple Summary:**

Oligodendrocyte (OD) is a cell type of great interest in the regenerative medicine for several neurological diseases. This study provides a new defined coating material for the differentiation of ODs from human pluripotent stem cells. A new peptide named VNP2, designed by in silico simulation, can be readily produced in a large amount and stably immobilized on the bottom of culture vessel. Upon using for differentiation of ODs, VNP2 promoted the differentiation efficiency more than the conventional coating materials did. Furthermore, transcriptomic analysis revealed molecular clues for the differentiation promoting activity of VNP2. Therefore, this peptide may be used as a favored coating material for the culture and differentiation of ODs.

**Abstract:**

Differentiation of oligodendrocytes (ODs) presents a challenge in regenerative medicine due to their role in various neurological diseases associated with dysmyelination and demyelination. Here, we designed a peptide derived from vitronectin (VN) using in silico docking simulation and examined its use as a synthetic substrate to support the differentiation of ODs derived from human pluripotent stem cells. The designed peptide, named VNP2, promoted OD differentiation induced by the overexpression of SOX10 in OD precursor cells compared with Matrigel and full-length VN. ODs differentiated on VNP2 exhibited greater contact with axon-mimicking nanofibers than those differentiated on Matrigel. Transcriptomic analysis revealed that the genes associated with morphogenesis, cytoskeleton remodeling, and OD differentiation were upregulated in cells grown on VNP2 compared with cells grown on Matrigel. This new synthetic VN-derived peptide can be used to develop a culture environment for efficient OD differentiation.

## 1. Introduction

Oligodendrocyte is a cell type in the central nervous system (CNS) that is responsible for the myelination of axons. This electrically inert insulation on neuronal axons is critical for the rapid conduction of nerve impulses. Thus, impairment or loss of oligodendrocyte function can cause various neurological diseases, including multiple sclerosis [1], leukodystrophy [2], and spinal cord injury [3]. Given their pivotal role in the pathophysiology of these diseases and their relevance in cell replacement therapy, oligodendrocyte development and regeneration has been investigated extensively over recent decades. Studies of rodent embryos have identified key genetic factors, including Olig2, a basic helix-loop-helix transcription factor [4], and sex-determining region Y-box 10 (Sox10) [5], and their network is critical for fate determination, specification, and maturation of oligodendrocytes during CNS development [6]. In addition, membrane-bound molecules that are specifically present on oligodendrocytes and their precursor cells (oligodendrocyte precursor cells, OPCs), such as NG2 chondroitin sulfate proteoglycan, O4, and the component of the myelin sheath, including myelin basic protein (MBP), have been used to identify and isolate them [7]. Furthermore, recent studies on the differentiation of OPCs and/or oligodendrocytes from human pluripotent stem cells (hPSCs), such as embryonic stem cells (hESCs) and induced pluripotent stem cells (hiPSCs), have substantiated the role of the microenvironment in the proper development of oligodendrocyte [8].

The extracellular matrix (ECM) is a key element of the microenvironment that provides both physico-mechanical and biochemical cues, which influence the cells’ proliferation, migration, and differentiation [9]. ECM components, such as vitronectin (VN), laminin, and thrombospondin, have been shown to promote oligodendrocyte differentiation during mouse development [10]. Molecular studies have revealed that these matrices facilitate oligodendrocyte differentiation through the interaction of integrins containing the αV subunit with the lysine-glycine-aspartate (RGD) motif on these ECM molecules [10]. In vitro studies have demonstrated that VN or a VN-derived peptide could promote the differentiation of human ESCs into OPCs [11,12]. Although these results elaborate on the role of VN in OPC differentiation, the involvement of VN in the maturation of oligodendrocytes remains largely unknown, and the molecular mechanisms underlying the impact of VN on the differentiation and maturation of oligodendrocytes are still elusive.

In the present study, we demonstrated that a new peptide originating from VN facilitates oligodendrocyte differentiation from hPSCs. Using in silico docking simulation, we designed several peptides from the VN sequence that have shown to support attachment and long-term culture of hPSCs. We compared the differentiation-promoting activity of these peptides with that of other conventional ECMs by culturing hPSC-derived OPCs overexpressing SOX10 on matrix-coated culture dishes. As a result, a specific VN peptide, named VNP2, was shown to differentiate oligodendrocytes from hPSC-derived OPCs more efficiently than other matrices, including Matrigel and full-length VN itself. The oligodendrocytes generated on VNP2 exhibited more processes in contact with the axon-mimicking nanofibers than those on the control matrix. Transcriptomic analysis revealed that the gene sets associated with morphogenesis and cytoskeleton remodeling were differentially expressed in both VN and VNP2 with respect to Matrigel. Cultivation of OPCs on VNP2 induced the expression of unique genes that are potentially involved in the differentiation and survival of oligodendrocytes, reflecting the differentiation-promoting activity of VNP2, distinct from VN. Our results substantiated the value of VN-derived peptides on the functional maturation of OPCs to oligodendrocytes and provide a new synthetic substrate for the generation of oligodendrocytes for future biomedical applications.

## 2. Materials and Methods

### 2.1. Designing New Peptide Motifs from Vitronectin

To design integrin-binding peptides from VN, a docking simulation was conducted using Autodock Vina. The VN peptide (KGGPQVTRGDVFTMP) and the crystal structure of integrin α (PDB ID: 1m1x) were used as the original ligand and target receptor, respectively. A genetic algorithm was employed to design strongly binding VN-derived peptides. Briefly, a mutant library of 1000 VN-derivatives was generated by introducing random mutations within the original VN peptide. Their 3D structures were predicted using FoldX [13], and their binding affinities were estimated. The five top-ranked peptides were selected for the next round of selection and 995 new mutants were generated from these five peptides. This process was iterated three times and was sufficient to generate new peptides with higher binding affinities. Consequently, two peptides were identified with binding affinities of −8.0 kcal/mol (KGGPQVTRGDYCTFP) and −7.8 kcal/mol (KGGPGVTRGDYFTFP) and were named VNP1 and VNP2, respectively. The estimated binding affinity between the original VN peptide and its receptor, integrin α, was −6.2 kcal/mol. The two peptides were evaluated for oligodendrocyte differentiation.

### 2.2. Production and Coating of Engineered Peptides

We created recombinants of VN peptides fused with the mussel adhesive protein (MAP), as described elsewhere [14]. Briefly, DNA constructs were designed to encode the fusion protein of MAP with decapeptide repeats of fb-1 at each fb-5 terminus [15] and predicted peptide motifs of vitronectin, connected from the C-terminus of MAP. Production and purification of the recombinant proteins were performed following a procedure conventional for recombinant proteins, as previously described [14]. For efficient presentation of peptide motifs to the cells, the recombinant peptides were immobilized on the culture vessel using the peptide-acrylate surface coating process [16]. Before coating the culture plate or nanofiber (see below) with the purified peptides, we activated multi-well plates or chambers with nanofibers by treating them with a solution containing 10 mM 1-ethyl-3-dimethylaminopropyl carbodiimide (EDC) (*AK Scientific*, San Francisco, CA, USA) and 10 mM N-hydroxy succinimide (NHS) (*AK Scientific*) in 20 mM sodium acetate buffer (pH 6.5) at room temperature for 30 min. After this, 0.05 µg/mL peptide solution was added to the activated vessels and incubated at room temperature for 30 min. The culture vessels were then washed three times with distilled water, completely dried, and stored at room temperature prior to use.

### 2.3. Culturing and Differentiation of Human ESCs

In this study, we used two hPSC lines, an ESC line (WA09, commonly known as H9) obtained from WiCell (Madison, WI, USA) and an iPSC line that we previously established (NL1) [17]. The majority of the differentiation data in this study were obtained with the ESC line (H9), unless indicated otherwise. hPSCs were cultured in feeder-free medium (StemMACS^®^ iPS-Brew XF) (Miltenyi Biotec, Bergisch Gladbach, Germany) on a Matrigel^®^ hESC-qualified matrix (Corning, NY, USA). For OPC differentiation, we adopted the protocol previously reported by Fossati et al. [18], with minor modifications. Briefly, hPSCs were seeded on Matrigel-coated plates at a density of 2.5 × 10^4^ cells/cm^2^ and cultured in neural induction medium (Supplementary Table S1) supplemented with 100 nM retinoic acid (RA) (Millipore Sigma, Burlington, MA, USA) for 12 days. 250 nM LDN193189 (Selleck Chemicals, Houston, TX, USA) and 10 µM SB431542 (Millipore Sigma) were added to the culture for the first 8 days to facilitate induction of the neuroectoderm. 1 µM Smoothened agonist (SAG) (Millipore Sigma) was added to the medium from day 8 to day 12 to commit the cells to OPC differentiation. The cells at day 12 of differentiation, which we referred to as OPCs once we checked immunoreactivity against OLIG2, were expanded by multiple passages in the OPC medium (Supplementary Table S1). For oligodendrocyte differentiation, OPCs (at passage 3–5 before cryopreservation) were seeded at a density of 2.0 × 10^5^ cells/cm^2^ on a culture plate pre-coated with various matrices in OPC medium. One day later, the cells were infected with a lentivirus encoding SOX10 (Addgene, clone ID number 45843, Watertown, MA, USA) and a lentivirus of reverse tetracycline-controlled transactivator (*rtTA*) (Addgene, clone ID number 20342) for doxycycline (DOX)-induced SOX10 expression and incubated for 14–16 h. Viral vectors and packaging were constructed as described elsewhere [17]. After incubation with viruses, the culture medium was replenished with fresh medium containing 2.5 µg/mL (DOX), included in the media for the entire differentiation period. The cells were then cultured in oligodendrocyte maturation medium (Supplementary Table S1) for two weeks. To examine the ability to respond to the nanofiber, differentiating oligodendrocytes were transferred onto the nanofiber (Millipore Sigma) 5 days after DOX induction at a density of 2.0 × 10^5^ cells/well and cultured for 5 to 7 days. Nanofibers were pre-coated with either Matrigel (at 1/10 dilution) or VNP2 (0.05 µg/mL) followed by Matrigel (at 1/60~1/90 dilution) for better attachment.

### 2.4. Immunofluorescent Staining

For live staining, the cells were washed once with DMEM/F12 (Thermo Fisher Scientific, Waltham, MA, USA) at 10–12 days of differentiation and replenished with fresh oligodendrocyte maturation media containing primary antibodies (mouse anti-O4 antibody, 1:400, R&D Systems, Minneapolis, MN, USA; and rabbit anti-MBP antibody, 1:100, Millipore Sigma). The cells were incubated with primary antibodies for 1 h in a CO_2_ incubator. After washing with DMEM/F12, secondary antibodies conjugated to the appropriate fluorescent dye (anti-rabbit Alex Fluor^®^ 568 or anti-mouse Alex Fluor^®^ 568, both from Thermo Fisher Scientific) were added and incubated for 30 min in a CO_2_ incubator. The cells were then washed with DMEM/F12 and replenished with fresh culture medium. Images of positive cells were captured using a fluorescence microscope (IX72; Olympus, Tokyo, Japan) equipped with a digital camera (DP72, Olympus). To obtain high-quality images, the cells on substrate-coated culture plates were transferred onto glass coverslips coated with the corresponding substrate. A day later, the samples were fixed with 4% paraformaldehyde (PFA)-phosphate buffered saline (PBS) solution for 10 min and mounted on glass slides using VECTASHIELD^®^ mounting medium containing 4′,6-diamidine-2′-phenylindole dihydrochloride (DAPI) (Vector Laboratories, Burlingame, CA, USA).

For general immunocytochemistry, the cells were fixed with 4% PFA-PBS solution for 10 min and permeabilized by treatment with 0.1% Triton X-100 in PBS for 10 min. The permeabilization step was omitted when targeting membrane-bound molecules. After blocking with 2% bovine serum albumin-PBS solution at room temperature for 1 h, the samples were incubated with primary antibodies overnight at 4 °C. The antibodies used in this study were: NESTIN (1: 200, Millipore Sigma), A2B5 (1:200, Millipore Sigma), NG2 (1:100, Millipore Sigma), NKX2.2 (1:200, DSHB, Iowa City, IA, USA), OLIG2 (1:100, R&D systems), O4 (1:400, R&D Systems), cleaved caspase-3 (1:4000, Cell Signaling, Danvers, MA, USA), Ki67 (1:2000, Leica Biosystems, Wetzlar, Germany), and MBP (1:100, Millipore Sigma). Unbound antibodies were washed away with PBS, and then secondary antibodies conjugated to a fluorescent dye were bound at room temperature for 1 h. After intensive washing, the samples were mounted on glass slides using mounting solution and cell images captured as described above.

### 2.5. Isolation of RNA and Quantitative Reverse Transcription Polymerase Chain Reaction (RT-PCR)

Total RNA was isolated using TRIzol ^®^ reagent (Thermo Fisher Scientific) following the manufacturer’s instructions. cDNA was synthesized from 1 µg of total RNA using PrimeScript^TM^ RT Master Mix (Takara, Japan). Real-time PCR for quantitative analysis of gene expression was conducted using Power SYBR^®^ Green PCR Master Mix (Thermo Fisher Scientific) and StepOnePlus^®^ real-time PCR system (Thermo Fisher Scientific). Expression values of specific markers were normalized to those of β-actin, and then the normalized expression was compared with expression in the control sample. The primer sequences used in this study are listed in Supplementary Table S2.

### 2.6. Acquisition and Analysis of RNAseq Data

Total RNA was isolated as described above and submitted to MACROGEN, Korea, for paired-end sequencing, with the aim of generating over 60 million reads. Adapter sequences were trimmed using TrimGalore [19], and transcript quantification was performed using Salmon [20], using the reference transcript GENCODEv32. To identify differentially expressed genes (DEGs), transcript abundance estimates were imported into DESeq2 [21] using tximport [22] for differential gene analysis. Differentially expressed genes are categorized using DEGreport (1.26.0) [23]. A correlation plot was generated using ggplot2 (v3.3.0) and a heatmap was generated using pheatmap (v1.0.12). Gene ontology analysis and protein-protein interaction (PPI) analysis was performed using Metascape [24].

### 2.7. Statistical Analysis

Relative expression levels are expressed as mean ± standard error of the mean. All experiments were performed at least three times to compute statistical significance. Experimental data were analyzed using Student’s *t*-test or one-way analysis of variance (ANOVA) for more than two experimental groups. Differences were considered statistically significant at a *p*-value < 0.05.

## 3. Results

### 3.1. Prediction and Identification of Novel Peptide Motifs from VN

**Figure 1:**
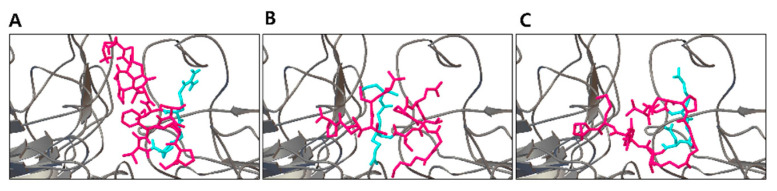
Docking poses of vitronectin peptide and designed peptides. The binding poses of vitronectin peptide (**A**), VNP1 (KGGPQVTRGDYCTFP) (**B**), and VNP2 (KGGPGVTRGDYFTFP) (**C**) are illustrated. The alpha and beta chains of integrin are shown in grey color. The three peptides are shown in red color and their RGD motifs are shown in cyan color.

An initial library of 1000 VN-derived mutants was generated by introducing random mutations into the VN peptide, but not in the RGD motif. The peptides in the library were then simulated to estimate their binding affinities to the binding pocket of integrin using Autodock Vina [25]. Five peptides with the strongest affinities were selected, and new mutations were introduced into these selected peptides to generate another library of 995 mutant peptides. After three rounds of mutation introduction and selection, the top two mutant peptides, KGGPQVT**RGD**YCTFP (−8.0 kcal/mol, VNP1) and KGGPGVT**RGD**YFTFP (−7.8 kcal/mol, VNP2) were selected. The RGD motif is shown in bold and amino acid residues different from the original VN peptide that were reported to efficiently support hPSC maintenance (KGGPQVTRGDVFTMP) [16] are underlined. The predicted 3D structures of the two mutant peptides and the original VN peptide are shown in Figure 1. The two designed peptides were experimentally evaluated for their oligodendrocyte differentiation capabilities.

### 3.2. Differentiation of OPCs from hPSCs

**Figure 2:**
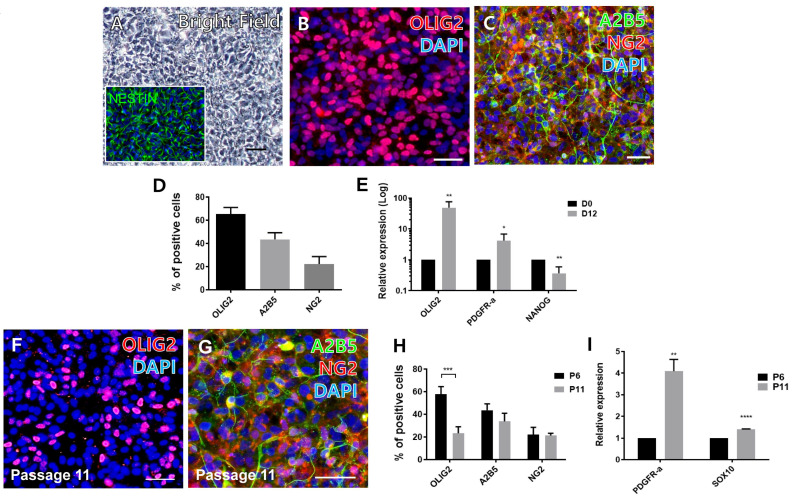
Induction and expansion of hPSC-derived OPCs. (**A**) A bright-field image of OPCs at day 12 of differentiation. Scale bar: 20 µm. (Inset) A representative image of NESTIN-positive cells in the culture on day 12. (**B**,**C**) Immunofluorescent images of OLIG2, A2B5 and NG2. (**D**) Quantification of cells positive for OLIG2, A2B5, and NG2. (**E**) Quantitative gene expression of OPC markers (*OLIG2* and *PDGFRα*) and a pluripotency marker (*NANOG*). (**F**,**G**) Representative images of OLIG2, A2B5- and NG2-positive cells after multiple passages (at passage 11) accompanied with a freeze-thawing cycle. Scale bar: 20 µm. (**H**) Quantification of marker-positive cells at passage 11. (**I**) Quantitative gene expression of OPC markers (*PDGFRα* and *SOX10*). Scale bars: 10 µm. * *p* < 0.05, ** *p* < 0.01, *** *p* < 0.001, **** *p* < 0.0001, Student *t*-test.

To generate a cell source for evaluating the designed peptides for oligodendrocyte differentiation, we established a method for generating OPCs from hPSCs. hESCs (H9) were differentiated into OPCs using a previously described method [18]. On day 12 of differentiation, the cells became confluent and exhibited a columnar shape with strong immunoreactivity for NESTIN (Figure 2A), indicating that the culture contained mostly neural progenitors. To determine whether the differentiated cells retained the characteristics of OPCs, immunocytochemical analysis using antibodies against various OPC markers including OLIG2, A2B5, and NG2, was performed. Quantitative analysis revealed that 65.44% ± 2.80%, 43.58% ± 2.84%, and 22.26% ± 3.18% among total cells were positive for OLIG2, A2B5, and NG2, respectively (Figure 2B–D), demonstrating that a substantial number of cells in the culture were induced into OPCs. Quantitative RT-PCR analysis also revealed a notable upregulation of OPC marker genes (*OLIG2* and *PDGFR-α*) with a concomitant downregulation of *NANOG*, a pluripotency marker (Figure 2E). We were also able to differentiate OLIG2-positive OPCs from hiPSCs at comparable efficiency (Supplementary Figure S1).

Next, we examined whether the resulting OPCs were expandable through multiple passages. In the OPC medium (Supplementary Table S1), the cells were stably expanded for 5–6 passages by enzymatic dissociation accompanied by ROCK inhibitor treatment. Furthermore, the OPCs were expandable for 5–6 passages after freezing and thawing. Expression of OPC markers was not significantly altered, except for *OLIG2* (Figure 2G,H), even with significant upregulation of *PDGFR-α* and *SOX10* five passages after the freeze-thaw cycle (see passage 6 vs. passage 11 in Figure 2I), indicating that the cells are proliferative while maintaining their characteristics as OPCs. Since these cells could be obtained in large amounts by multiple passaging, we used them as a cell source to test the ability of VN-derived peptides to support the differentiation of oligodendrocytes.

### 3.3. Differentiation of OPCs into Oligodendrocytes through Forced Expression of SOX10

**Figure 3:**
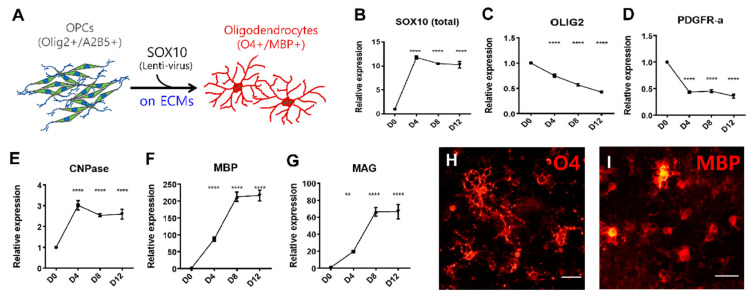
Differentiation of oligodendrocytes from hPSC-derived OPCs via SOX10 overexpression. (**A**) A schematic diagram for oligodendrocyte differentiation from hPSC-derived OPCs. (**B**–**D**) Time-course expression of OPC markers during differentiation. As differentiation proceeded, the transcript levels of OLIG2 and PDGFR-α gradually decreased (**C**,**D**) whereas that of SOX10 was maintained due to continued exogenous expression (**B**). (**E**–**G**) Time-course expression of OD markers during differentiation. Expression levels of all marker genes tested were upregulated by SOX10-induced differentiation and plateaued after day 8. (**H**,**I**) Representative image of O4- and MBP-positive cells induced by the overexpression of SOX10. Scale bars: 12.5 µm. ** *p* < 0.01, **** *p* < 0.0001, one-way ANOVA with Dunnett’s *post hoc* test.

Differentiation of OPCs into oligodendrocytes is a lengthy process. During embryonic development in mice, Olig2-positive OPCs appear at the early stages of the ventral spinal cord and the ventral forebrain formation and maintain their immature state until birth, whereas maturation for myelination mainly occurs postnatally in response to the activity of neuronal circuit [26,27,28]. Such long periods have also been witnessed in previous in vitro oligodendrocyte differentiation studies: protocols took months to generate mature oligodendrocytes from hPSCs and often required multiple culture steps with long-term OPC expansion in suspension [29,30]. This long culture period makes such protocols inappropriate for use as a platform for studying developmental mechanisms, especially for the impact of ECM molecules on the maturation of oligodendrocytes. A recent seminal study tackled this issue by providing a rapid differentiation strategy, in which the forced expression of SOX10 in OPCs generated MBP-positive oligodendrocytes within 3 weeks [31]. Since this approach induces maturation of OPCs while they are adhered to the culture dish, we adopted this method to examine the effect of VN-derived peptides on the maturation of OPCs into oligodendrocytes (Figure 3A).

We introduced SOX10 in OPCs using a DOX-inducible lentiviral system [17]. To optimize gene transduction, various titers of EGFP-lentivirus were infected. As shown in Supplementary Figure S2, a higher multiplicity of infection (MOI) (>4) was efficient in delivering the transgene, but resulted in significant cell death; thus, we determined to use an MOI of 2. We verified the reliability of SOX10-mediated differentiation of OPCs on Matrigel-coated culture dishes. Once DOX was added, the expression of *SOX10* rapidly increased while the levels of *OLIG2* and *PDGFRα* expression progressively declined over the course of differentiation (Figure 3B–D). Inversely, transcription levels of oligodendrocyte markers such as *CNPase*, *MBP*, and *MAG* gradually increased (Figure 3E–G). When examined by live staining with antibodies against O4 and MBP after DOX treatment on days 10–12, numerous cells were found to be positive for these markers with a typical morphology of oligodendrocytes (Figure 3H,I). This result confirms the reliability of the differentiation method for testing various substrates.

### 3.4. Screening of Various Substrates for Oligodendrocyte Differentiation

**Figure 4:**
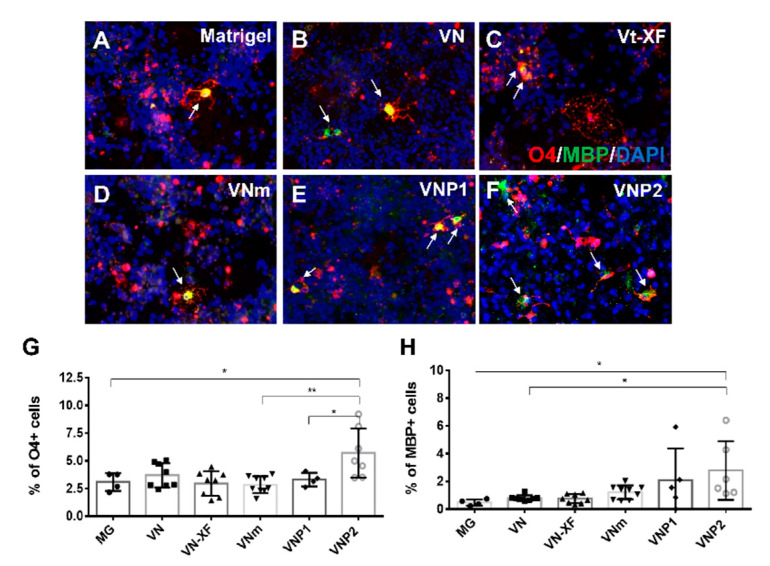
Screening for ECM materials facilitating oligodendrocyte differentiation induced by SOX10. (**A**–**F**) Representative images of O4- and MBP-positive cells generated on the indicated matrix. White arrows are pointing to double-positive cells for O4 and MBP. (**G**,**H**) Quantification of the number of O4- or MBP-positive cells on day 12 after initiation of oligodendrocyte maturation on the indicated substrates. * *p* < 0.05, ** *p* < 0.01, one-way ANOVA with Tukey’s *post-hoc* test.

We tested Matrigel, recombinant VN (50 µg/mL), commercially available VN-derived peptide (VN XF^®^ from STEMCELL Technologies), VNP1, and VNP2 to determine whether these ECM molecules support oligodendrocyte differentiation from hPSC-derived OPCs. Another VN-derived peptide (VNm) was also included in the list because we have previously shown that it can support stable adherence and long-term culture of hPSCs [32]. OPCs were seeded on various matrices at the same density and induced to differentiate through SOX10 overexpression. Quantitative gene expression analysis performed 2 days post-treatment with DOX revealed that *SOX10* expression level was not significantly different across the experimental groups (Supplementary Figure S3). Ten days later, small, and oval-shaped cell bodies with multiple processes were observed on all ECMs. Live staining revealed that the cells with such morphology exhibited immunoreactivity for O4, and occasionally for MBP, in all test groups (Figure 4A–F). Quantitative analysis revealed that 3.09% ± 0.40%, 3.68% ± 0.38%, 2.94% ± 0.39%, 3.30% ± 0.30%, 2.83% ± 0.25% of the total cells were positive for O4 on Matrigel, full-length vitronectin, VN XF^®^, VNP1, and VNm, respectively (Figure 4G). The differentiation efficiency was not significantly different across the groups. However, significantly more O4-positive cells (5.70% ± 0.84% of total) were detected on VNP2 than on Matrigel (Figure 4G). In the quantification of MBP-positive cells, more MBP-positive cells were detected when differentiated on VN or VN-derived peptides than on Matrigel (Figure 4H). Although the difference was not statistically significant, this result was consistent with previous findings that VN promotes oligodendrocyte differentiation [11]. More importantly, we observed a significantly higher number of MBP-positive cells on VNP2 than on Matrigel (3.2 folds, *p* = 0.015, one-way ANOVA, Tukey’s *post-hoc* test) (Figure 4H). Significant differences were also observed when the number of MBP-positive cells was compared between the VNP2 and full-length VN groups (*p* = 0.047, one-way ANOVA, Tukey’s *post-hoc* test) (Figure 4H). Significantly higher number of MBP-positive cells was also obtained on VNP2 than on Matrigel when hiPSC-derived OPCs were differentiated (Supplementary Figure S4), verifying the effectiveness of VNP2 on oligodendrocyte differentiation. To exclude the possibility that VNP2 facilitates oligodendrocyte differentiation by selectively attaching OPCs or inducing cell death in non-OPCs, we assessed cell attachment to VNP2 and Matrigel. As shown in Supplementary Figure S5, the number of OLIG2-positive cells examined 2 days after seeding was not significantly different between the two groups. In addition, immunostaining for Ki67 (a cell proliferation marker) and cleaved caspase-3 (an apoptotic marker) revealed that VNP2 neither selectively proliferated OPCs nor induced cell death compared with Matrigel (Supplementary Figure S5D–I). These results were further supported by a colorimetric assay (CCK-8 assay) that examined cell proliferation and cytotoxicity at various concentrations of VNP2 and Matrigel (Supplementary Figure S6). Our result suggests that the facilitated differentiation of oligodendrocytes on VNP2 was not a result of selective attachment, proliferation, or survival effect.

### 3.5. Differentiation of Oligodendrocytes on Nanofibers Coated with Matrigel or VNP2

**Figure 5:**
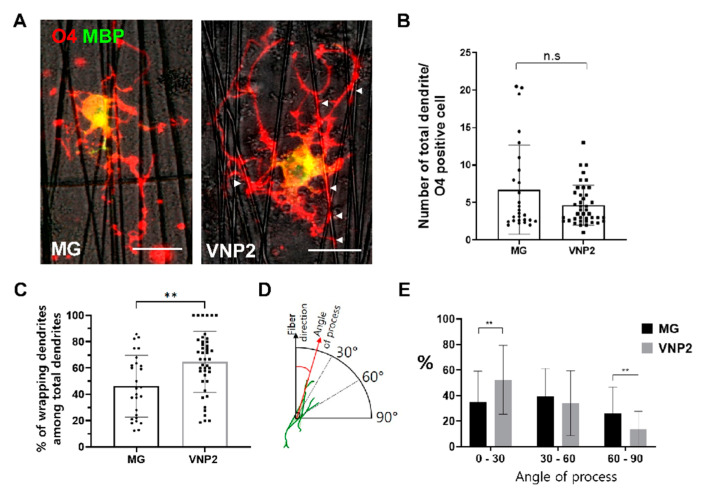
Behavior of oligodendrocytes differentiated on either Matrigel or VNP2 on nanofibers coated with corresponding substrates. (**A**) Representative images of O4- and MBP-positive cells differentiated on nanofibers coated with Matrigel or VNP2. Arrow heads point to the processes contacting with nanofibers. (**B**) Quantification of total processes per an O4-positive cell differentiated on nanofibers coated with the indicated substrate. (**C**) Quantification of processes contacting with nanofibers per an O4-positive cell on nanofibers coated with the indicated substrate. Note that O4-positive cells differentiated on VNP2-coated nanofibers exhibit more processes that contact with nanofibers. (**D**) A schematic diagram illustrating how to measure the angle of processes with respect to the direction of nanofibers. (**E**) Quantification of process numbers according to their angle with respect to the direction of nanofibers. Scale bars: 10 µm. ** *p* < 0.01, Student *t*-test.

The cell-free electrospun nanofiber is an excellent system for testing the functionality of oligodendrocytes, in which seeded oligodendrocytes align their processes along the length of nanofibers and ensheath them, mimicking in vivo axonal myelination [33]. Taking advantage of its convenience in testing the functionality of oligodendrocytes, we examined how differently oligodendrocytes behave on nanofibers with different coatings. We differentiated OPCs on either Matrigel or VNP2-coated plates for 5~6 days and transferred them to nanofibers coated with the corresponding substrates. Immunocytochemical staining performed 5 days after transfer showed that O4 and MBP double-positive cells were positioned between nanofibers and extended their processes toward them (Figure 5A). Quantification of the total number of processes did not reveal a significant difference between Matrigel and VNP2 (Figure 5B). However, when the percentage of processes overlapping nanofibers per O4-positive cells (indicated by arrowheads in Figure 5A) was measured, we observed that the cells on VNP2 had more nanofiber-contacting processes than those on Matrigel (Figure 5C). Consistently, when the angle of process with respect to the direction of nanofibers was examined (Figure 5D), we found that the cells on VNP2 had more processes that made acuter angles (0–30°) with nanofibers than those on Matrigel (Figure 5E). Although we did not obtain direct evidence for the ensheathment of nanofibers by oligodendrocytes, this result shows that oligodendrocytes on VNP2-coating were more intimately engaged with nanofibers that provide biophysical cues for ensheathment than those on Matrigel. Thus, our results suggest that VNP2 not only improves the differentiation efficiency of oligodendrocytes, but also facilitates the ability of cells to connect with a biophysical cue mimicking neuronal axons.

### 3.6. Differential Gene Expression in OPCs Differentiated on Different Matrices

**Figure 6:**
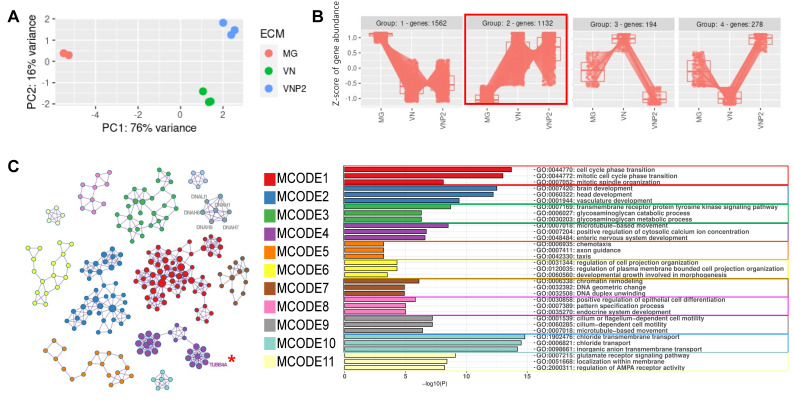
Analyses of DEGs among OPCs grown on Matrigel, VN, and VNP2. RNAseq samples were obtained from different cells grown on Matrigel, VN, or VNP2. (**A**) PCA analysis results of nine experimental samples (three for each growth condition). (**B**) Genes were grouped based on their expression patterns. Since group 2 was of interest (genes upregulated in VN and VNP2 samples), 1132 genes were analyzed further. (**C**) Genes in group 2 in (**B**) were used to identify core clusters in the PPI network. The identified clusters and their corresponding GO terms are shown.

To gain insight into the molecular mechanism underlying VNP2 function in oligodendrocyte differentiation, comparative analysis was performed using transcriptomic data (RNAseq) obtained from OPCs grown on either VNP2, VN, or Matrigel for two days without SOX10 overexpression. SOX10 overexpression was excluded because it could have a more marked impact on global gene expression than the effect of matrices. We included VN because, despite the differentiation-promoting effect of VN on oligodendrocyte differentiation, its molecular mechanism has not yet been elucidated [11]. Principal component analysis (PCA) results showed clear clustering of the samples with respect to the substrate used (Figure 6A), indicating sufficient reliability of the transcriptomic data for further analysis and different impacts of each substrate on the transcriptome. The heat map for the expression of the cell fate-related genes demonstrated a clear upregulation of OPC marker genes with a downregulation of genes involved in the other neural fates (i.e., neurons and astrocytes) as well as pluripotency in all groups (Supplementary Figure S7A), verifying that the cell culture was indeed enriched in OPCs.

Next, DEGs with a likelihood ratio test (LRT) (p_adj_ < 0.05) were identified across the three substrates. The DEGs were grouped based on their expression patterns using DEGreport package (Figure 6B). Although four clusters were identified, the two largest groups (group 1 and 2) accounted for 85% (1562 and 1132 genes) of the DEGs. This indicates that VN and VNP2 share a similar gene expression profile compared with Matrigel. Of the groups, the genes upregulated in VN and VNP2 versus Matrigel (group 2 in the red box in Figure 6B) stood out, which may explain the different effects of VN and VNP2 on oligodendrocyte differentiation from Matrigel. Moreover, group 2 included transcription factors essential for oligodendrocyte differentiation, such as *OLIG1*, *OLIG2*, and *NKX2.2* (Supplementary Figure S7B–D). It should also be noted that the expression of *OLIG1* and *NKX2.2* was highest in VNP2. Therefore, we performed Gene Ontology (GO) enrichment analysis (Supplementary Figure S8) and protein–protein interaction (PPI) network analysis (Figure 6C) with the DEGs belonging to group 2. The results revealed that genes involved in cell cycle (MCODE1) and development (MCODE2) were highly enriched within the DEGs in group 2 (Figure 6C), which is in agreement with previous results showing molecular signature changes during rodent OPC differentiation from neonatal to postnatal stages [34]. In addition, enrichment of genes associated with morphogenesis (MCODE6), and microtubule-based movement (MCODE4 and MCODE9) was noticeable. This result indicates that VN and VNP2 may induce expression of the genes required for drastic morphological changes. The other interesting function enriched in the cells grown on VN or VNP2 compared with those grown on Matrigel was chloride channel (MCODE10) and glutamate receptor activity (MCODE11) (Figure 6C). It has recently been reported that chloride channels and glutamate receptors are involved in oligodendrocyte differentiation and myelination [35,36]. Consequently, VN and VNP2 are more able to upregulate the expression of genes favorable for oligodendrocyte differentiation and myelination in OPCs than in Matrigel.

**Figure 7:**
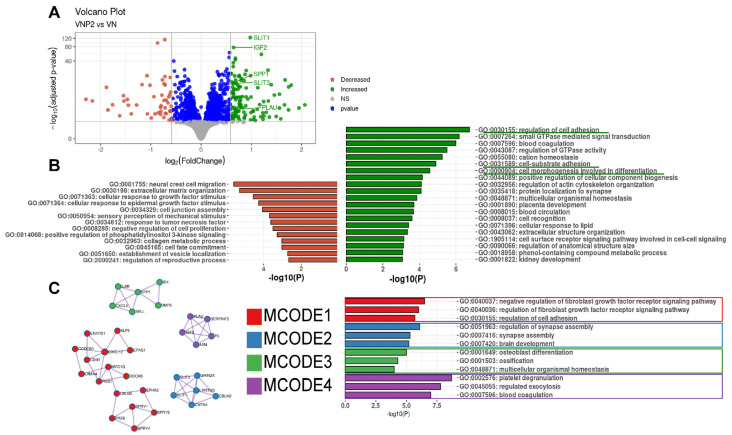
Analysis of genes that are differentially expressed between OPCs grown on VN and VNP2. (**A**) A volcano plot of genes obtained from OPCs grown on either VN or VNP2. Dots in green color denote upregulated genes in VNP2, and dots in red color denote downregulated genes in VNP2 compared with VN. (**B**) GO terms enriched within downregulated genes (red) and upregulated genes (green). (**C**) Since no significant clusters were identified in the downregulated genes, only the clusters identified in the upregulated genes are shown.

To further investigate the difference between VN and VNP2 on the promotion of oligodendrocyte differentiation, DEGs were identified between VN and VNP2 (Figure 7A) and GO enrichment and PPI network analyses were performed (Figure 7B,C). The downregulated genes (red dots in Figure 7A and red bars in Figure 7B) were involved in neural crest cell migration (GO:0001755), cellular response to growth factor stimulus (GO: 0071363 and GO: 0071364), and organization of extracellular components (GO: 0030198). In contrast, the upregulated genes in VNP2 were involved in the regulation of cell adhesion (GO: 0030155), cell-substrate adhesion (GO: 0031589), and cell morphogenesis involved in differentiation (GO: 0000904) (indicated by green lines in Figure 7B). A detailed list of the DEGs is presented in Supplementary Table S3. In the PPI network analysis, the downregulated genes did not show any significant functional clusters. The clustered genes identified within the upregulated genes are shown in Figure 7C. The largest cluster identified was composed of proteins involved in the negative regulation of FGFR-mediated signaling. This is consistent with previous results, which showed that the inhibition of FGF signaling has a positive effect on oligodendrocyte differentiation [37]. Thus, negative regulation of FGFR-mediated signaling may be one of the potential mechanisms underlying the oligodendrocyte-promoting activity of VNP2 over VN. We also found supporting evidence in the literature on the association among the proteins included in the clusters with oligodendrocyte differentiation. Specifically, secreted phosphoprotein 1 (SPP1) in MCODE3 is a glycoprotein that influences the proliferation, differentiation, and survival of multiple cell types, including Schwann cells [38]. Insulin-like growth factor-2 (IGF-2) in MCODE4 protects oligodendrocytes from cell death via neuroinflammation [39]. A urokinase-type plasminogen activator (PLAU) in MCODE4 was expressed by oligodendrocytes in the developing rat brain [40]. Therefore, it is plausible that the upregulation of these genes is beneficial for the survival and/or differentiation of oligodendrocytes. Proteins inhibiting axonal projection (e.g., SLIT1 and SLIT3 in MCODE2) were also upregulated in the VNP2 group, consistent with the fact that oligodendrocytes can inhibit axonal growth [41]. In addition, previous studies have demonstrated the inhibitory role of SLITs in axonal projection in *Drosophila* midline glial cells, similar to mammalian oligodendrocytes [42,43]. Upregulation of these genes may provide indirect evidence for the differentiation promoting effect of VNP2 over VN.

Together, our results demonstrate that the cultivation of OPCs on either VN or VNP2 enhances the efficiency of oligodendrocyte differentiation by inducing common genes involved in oligodendrocyte differentiation. Furthermore, VNP2 further increases efficiency compared with VN by suppressing differentiation toward the other lineage (i.e., neural crest) and triggering the expression of additional genes potentially involved in the survival and differentiation of oligodendrocytes.

## 4. Discussion

The use of ECMs in the differentiation of stem cells has been substantiated in the last decade. ECMs affect the survival, growth, and fate decisions of stem cells by providing critical biological and physiochemical environments. To date, various ECM molecules have been identified for diverse biomedical applications, specifically to direct the differentiation of stem cells [44]. VN is one of the most widely used ECMs, particularly for hPSC engineering, because VN and VN-derived peptides are capable of maintaining hPSCs [16,45] and even facilitating their differentiation into specific cell types, including oligodendrocytes [32]. In this study, we to create a novel peptide derived from the VN peptide for the differentiation of oligodendrocytes. For rational design, we searched for peptide candidates that showed strong binding affinities for integrin (receptor for VN) using in silico docking, an attractive method used to identify molecular ligands solely based on 3D structures. Such structure-based approaches have been widely used in drug discovery and peptide/protein engineering [46,47]. As a result, two VN-derived peptides with higher binding affinities than VN were designed: KGGPQVTRGDYCTFP (VNP1) and KGGPGVTRGDYFTFP (VNP2). Since both VNP1 and VNP2 contain the RGD motif that is critical for integrin binding, they could support the adhesion of OPCs through direct interaction with integrin. The designed peptides were likely able to interact with integrin in a manner similar to VN, as our transcriptomic analysis showed that OPCs grown on either VN or VNP2 largely shared similar gene expression patterns.

Importantly, our transcriptomic analysis illustrates a molecular basis for the ability of VN and VNP2 to promote oligodendrocyte differentiation. Noticeably, GO terms associated with morphogenesis and microtubule (MT)-based movement were enriched within DEGs of cells grown on VN and VNP2. Given that oligodendrocytes exhibit prominent complexity with highly ramified processes, it is not surprising that massive rearrangement of cytoskeletons occurs during the differentiation of OPCs into oligodendrocytes. Thus, it is possible that the enrichment of genes involved in such morphological changes significantly contributes to the increased efficiency of oligodendrocyte differentiation on VN and VNP2. In particular, MT is the primary component of oligodendrocyte cytoskeleton that provides the mechanical stability needed for OD processes and serves as a ‘highway’ for transportation [48], and thereby plays a key role in oligodendrocyte morphogenesis, including process extension, contact with axons, and myelin compaction [49]. Among various subtypes of β-tubulin, one of the components forming MT, β_IV_-tubulin is exclusively produced in oligodendrocytes and implicated in hypomyelinating leukodystrophy [50,51]. Unexpectedly, the expression of *TUBB4A,* a gene encoding β_IV_-tubulin, was upregulated in cells grown on VN and VNP2 compared with that in cells grown on Matrigel (indicated by an asterisk in Figure 6C). This suggests that VN or VNP2 instructs OPCs to express a particular MT component specific for oligodendrocytes, such that the cells reorganize their cytoskeleton in favor of differentiation into oligodendrocytes. In addition, genes, such as dynein (e.g., *DNAH1*, *DNAH6*, *DNAH7*, *DNAH9*, and *DNALI1*) involved in MT-based movement (transportation) were also upregulated in the VN and VNP2 groups versus the Matrigel group. Collectively, these results suggest that the facilitation of MT-mediated cytoskeleton remodeling is a common mechanism underlying enhanced oligodendrocyte differentiation by VN and VNP2.

Finally, the analysis of DEGs between VN and VNP2 provides molecular insights into how VNP2 outperforms VN in oligodendrocyte differentiation. First, the upregulated cellular functions associated with the differentiation and function of oligodendrocytes in VNP2 may contribute to expediting the differentiation of OPCs. Second, higher expression of *OLIG1* and *NKX2.2* on VNP2 relative to VN may facilitate the differentiation of OPCs into oligodendrocytes. This possibility is supported by previous studies showing that Olig1 plays a critical role in the differentiation of OPCs into oligodendrocytes and in the subsequent myelination in developing mice [52]. Furthermore, Nkx2.2, in collaboration with Olig2, promotes oligodendrocyte differentiation in chick embryos [4]. Consequently, our results suggest that VNP2 retains VN’s ability to induce oligodendrocyte differentiation and acquires additional ability to enhance the efficiency of differentiation.

Although there was a significant improvement in OD differentiation, the activity of VNP2 was only tested in the differentiation involving SOX10 overexpression; thus, it needs to be further evaluated using other differentiation methods. In addition, the low yield of O4-positive cells (5.7% among total cells, on average) obtained upon using VNP2 presents a limitation of the current approach. Despite the significant increase in comparison with Matrigel (3.2 folds), this level of efficiency and purity may not be acceptable for large-scale production for biomedical applications. This might be due to the suboptimal culture conditions used in this study; in the previous study, SOX10-mediated differentiation resulted in an O4-positive cell yield of approximately 55% [31]. Thus, optimization of differentiation conditions may help enhance the usability of VNP2 for OD differentiation.

In conclusion, we designed a new VN-derived peptide (VNP2) in this study that promotes the differentiation of oligodendrocytes from hPSC-derived OPCs. Since this peptide can be synthesized and immobilized on a culture plate completely devoid of materials originating from animals, the oligodendrocyte differentiated on this substrate may be a favored cell source for future biomedical applications.

## Data Availability

The data that supports the findings of this study are available in the supplementary material of this article. All FASTQ files and Supplementary files were uploaded to National Center for Biotechnology Information Gene Expression Omnibus under accession code GSE173826.

## Supplementary Materials

https://www.mdpi.com/article/10.3390/biology10121254/s1. Figure S1: Differentiation of OPCs from hiPSCs (NL1 line), Figure S2: Optimizing virus titers for gene transduction, Figure S3: Comparison of the levels of SOX10 expression in OPCs infected with SOX10-lentivirus on various substrates, Figure S4: Differentiation of oligodendrocytes from hiPSCs (NL1 line), Figure S5: Comparison of the attachment, proliferation, and survival of OPCs on Matrigel vs. VNP2, Figure S6: Examination of cell attachment at various concentrations of Matrigel and VNP2, Figure S7: Comparison of the expression of transcription factors critical for oligodendrocyte differentiation on Matrigel, VN, and VNP2, Figure S8: GO term enrichment analysis for significantly changed genes in OPCs grown on Matrigel vs. VN and VNP2, Table S1: Formula of culture media used in this study, Table S2: Primer sequences used for quantitative RT-PCR, Table S3: DEGs list (VNP2 vs. VN).
