# Supplementary material for: A Novel Vitronectin Peptide Facilitates Differentiation of Oligodendrocytes from Human Pluripotent Stem Cells (Synthetic ECM for Oligodendrocyte Differentiation)
Source: Biology (Basel). 2021 Dec 1;10(12):1254. doi: 10.3390/biology10121254 (PMC8698880; doi:10.3390/biology10121254)
Supplement: Supplementary file 1 [file biology-10-01254-s001.zip › biology-1231138-Supplementary Materials.pdf]

**Table S1. Formula of culture media used in this study**

| Media | Components | Provider | Final concentration |
|---|---|---|---|
| Neural induction medium | DMEM/F12 | Thermo Fisher | |
| | Glutamax (100X) | Thermo Fisher | 1X |
| | Non-Essential Aminoacids(100X) | Thermo Fisher | 1X |
| | Beta-Mercaptoethanol (1000X) | Thermo Fisher | 1X |
| | Penicillin-Streptomycin (100X) | Thermo Fisher | 1X |
| | N2 supplement (100X) | Thermo Fisher | 1X |
| | B27 supplement (50X) | Thermo Fisher | 1X |
| | SB431524 | Sigma Millipore | 10uM |
| | LDN193189 | Selleck chemical | 250nM |
| OPC medium | DMEM/F12 | Thermo Fisher | |
| | Glutamax (100X) | Thermo Fisher | 1X |
| | Non-Essential Aminoacids(100X) | Thermo Fisher | 1X |
| | Beta-Mercaptoethanol (1000X) | Thermo Fisher | 1X |
| | Penicillin-Streptomycin (100X) | Thermo Fisher | 1X |
| | N2 supplement (100X) | Thermo Fisher | 1X |
| | B27 supplement (50X) | Thermo Fisher | 1X |
| | Retinoic acid (RA) | Sigma Millipore | 100nM |
| | Smoothened agonist (SAG) | Sigma Millipore | 1μM |
| | Insulin | Sigma Millipore | 25ug/ml |
| | Basic fibroblast growth factor | Prospec | 20ng/ml |
| OD maturation medium | DMEM/F12 | Thermo Fisher | |
| | Glutamax (100X) | Thermo Fisher | 1X |
| | Non-Essential Aminoacids(100X) | Thermo Fisher | 1X |
| | Beta-Mercaptoethanol (1000X) | Thermo Fisher | 1X |
| | Penicillin-Streptomycin (100X) | Thermo Fisher | 1X |
| | N2 supplement (100X) | Thermo Fisher | 1X |
| | B27 supplement (50X) | Thermo Fisher | 1X |
| | Insulin | Sigma Millipore | 25μg/ml |
| | Biotin | Sigma Millipore | 100ng/ml |
| | T3 | Sigma Millipore | 60ng/ml |
| | PDGF-AA | Prospec | 10ng/ml |
| | IGF-1 | Prospec | 10ng/ml |
| | HGF | Pepprotech | 5ng/ml |
| | NT3 | Prospec | 10ng/ml |
| | cAMP | Sigma Millipore | 1μM |

**Table S2. Primer sequences used for quantitative RT-PCR**

| Primer | Forward (5' – 3') | Reverse (5' – 3') |
|---|---|---|
| Beta actin | CAC CAT TGG CAA TGA GCG GTT C | AGG TCC TTG CGG ATG TCC ACG T |
| Olig2 | CGA CTC ATC TTT CCT TCT CTA A | CGC ACT TAC CTC ATC ATT G |
| PDGFR-a | GAC TTT CGC CAA AGT GGA GGAG | AGC CAC CGT GAG TTC AGA ACG C |
| Sox10 | ATG AAC GCC TTC ATG GTG TGG G | CGC TTG TCA CTT TCG TTC AGC AG |
| Nanog | CTC CAA CAT CCT GAA CCT CAG C | CGT CAC ACC ATT GCT ATT CTT CG |
| MBP | AGG ATT TGG CTA CGG AGG CAG A | GGT TTT CAG CGT CTA GCC ATG G |
| CNPase | CTG CTA GAG TGC AAG ACG CTC | GTG CCA TCA CGG TAC TTG TCC |
| MAG | GCT CAG TGT CAT GTA TGC ACC | GGA GCA CAA GAT AGA GAC CGT |

**Table S3. DEGs list (VNP2 vs VN)**

| ensembl | baseMean | log2FoldChange | lfcSE | pvalue | padj | symbol |
|---|---|---|---|---|---|---|
| ENSG00000120907 | 37.05883 | 2.063729 | 0.504234 | 1.65E-06 | 9.32E-05 | ADRA1A |
| ENSG00000186105 | 31.90858 | 1.945468 | 0.534636 | 1.05E-05 | 0.000467 | LRRC70 |
| ENSG00000188404 | 150.2998 | 1.791755 | 0.241054 | 5.02E-15 | 1.14E-12 | SELL |
| ENSG00000074803 | 136.1073 | 1.741252 | 0.245615 | 6.32E-14 | 1.29E-11 | SLC12A1 |
| ENSG00000125618 | 36.75181 | 1.73868 | 0.524265 | 2.85E-05 | 0.00111 | PAX8 |
| ENSG00000198734 | 215.6007 | 1.67048 | 0.194419 | 7.28E-19 | 2.73E-16 | F5 |
| ENSG00000160712 | 70.69187 | 1.658494 | 0.411129 | 1.62E-06 | 9.16E-05 | IL6R |
| ENSG00000250273 | 23.70306 | 1.584532 | 1.075776 | 0.001348 | 0.026124 | PSMC1P5 |
| ENSG00000124208 | 146.8774 | 1.558451 | 0.271779 | 3.30E-10 | 4.15E-08 | TMEM189-UBE2V1 |
| ENSG00000164972 | 48.67723 | 1.557692 | 0.430608 | 1.10E-05 | 0.000478 | C9orf24 |
| ENSG00000164619 | 28.54851 | 1.494197 | 0.640745 | 0.000504 | 0.012347 | BMPER |
| ENSG00000135218 | 133.291 | 1.455898 | 0.259236 | 7.65E-10 | 8.93E-08 | CD36 |
| ENSG00000145244 | 251.8052 | 1.394978 | 0.185725 | 2.65E-15 | 6.28E-13 | CORIN |
| ENSG00000108950 | 32.23199 | 1.358788 | 0.627946 | 0.00067 | 0.015395 | FAM20A |
| ENSG00000182732 | 122.1529 | 1.337888 | 0.286857 | 1.17E-07 | 8.55E-06 | RGS6 |
| ENSG00000188803 | 537.0906 | 1.32986 | 0.121458 | 3.08E-29 | 3.31E-26 | SHISA6 |
| ENSG00000172137 | 51.1587 | 1.295019 | 0.428389 | 8.81E-05 | 0.002861 | CALB2 |
| ENSG00000183631 | 39.36058 | 1.249127 | 0.512672 | 0.000423 | 0.01084 | PRR32 |
| ENSG00000175899 | 145.5523 | 1.244468 | 0.271871 | 1.78E-07 | 1.26E-05 | A2M |
| ENSG00000284431 | 30.59085 | 1.231623 | 0.689587 | 0.001555 | 0.028977 | NA |
| ENSG00000154330 | 54.02707 | 1.226512 | 0.422122 | 0.000122 | 0.003716 | PGM5 |
| ENSG00000044524 | 1549.422 | 1.198752 | 0.074567 | 1.86E-59 | 4.99E-56 | EPHA3 |
| ENSG00000125810 | 41.18496 | 1.188155 | 0.518152 | 0.000568 | 0.013585 | CD93 |
| ENSG00000007129 | 115.9785 | 1.164146 | 0.282661 | 1.43E-06 | 8.28E-05 | CEACAM21 |
| ENSG00000108684 | 317.0688 | 1.127467 | 0.162887 | 1.91E-13 | 3.57E-11 | ASIC2 |
| ENSG00000151655 | 86.43641 | 1.11579 | 0.321603 | 2.03E-05 | 0.000823 | ITIH2 |
| ENSG00000090104 | 30.33757 | 1.081204 | 0.71022 | 0.002464 | 0.041142 | RGS1 |

| | | | | | | |
|---|---|---|---|---|---|---|
| ENSG00000125089 | 47.72176 | 1.055939 | 0.468055 | 0.000675 | 0.015458 | SH3TC1 |
| ENSG00000272410 | 49.71654 | 1.045088 | 0.881999 | 0.001935 | 0.034312 | RP11-438J1.1 |
| ENSG00000181649 | 116.0026 | 1.03633 | 0.32477 | 4.24E-05 | 0.001534 | PHLDA2 |
| ENSG00000105479 | 83.13028 | 1.033783 | 0.326489 | 5.55E-05 | 0.001921 | CCDC114 |
| ENSG00000111432 | 85.01367 | 0.991773 | 0.334871 | 0.000103 | 0.00328 | FZD10 |
| ENSG00000172164 | 236.9085 | 0.991715 | 0.185878 | 4.40E-09 | 4.38E-07 | SNTB1 |
| ENSG00000134215 | 755.6642 | 0.983005 | 0.103279 | 8.97E-23 | 4.74E-20 | VAV3 |
| ENSG00000273184 | 42.2666 | 0.982758 | 0.595173 | 0.001921 | 0.03419 | RP11-212P7.3 |
| ENSG00000187122 | 12180.55 | 0.977697 | 0.040586 | 7.96E-130 | 1.28E-125 | SLIT1 |
| ENSG00000269113 | 255.7326 | 0.929332 | 0.185801 | 2.65E-08 | 2.21E-06 | TRABD2B |
| ENSG00000174640 | 491.3589 | 0.898514 | 0.130572 | 2.87E-13 | 5.25E-11 | SLCO2A1 |
| ENSG00000069535 | 1154.378 | 0.897886 | 0.082702 | 9.02E-29 | 9.08E-26 | MAOB |
| ENSG00000108231 | 299.602 | 0.894894 | 0.167008 | 3.83E-09 | 3.93E-07 | LGI1 |
| ENSG00000034239 | 169.93 | 0.888188 | 0.221152 | 2.95E-06 | 0.000154 | EFCAB1 |
| ENSG00000077943 | 92.81062 | 0.883718 | 0.338725 | 0.000277 | 0.007617 | ITGA8 |
| ENSG00000112769 | 1374.206 | 0.873282 | 0.088605 | 3.19E-24 | 2.14E-21 | LAMA4 |
| ENSG00000187800 | 73.83669 | 0.863186 | 0.38744 | 0.00072 | 0.016119 | PEAR1 |
| ENSG00000187783 | 75.65855 | 0.857253 | 0.425024 | 0.001094 | 0.022107 | TMEM72 |
| ENSG00000107562 | 810.1285 | 0.856336 | 0.102074 | 2.48E-18 | 8.32E-16 | CXCL12 |
| ENSG00000257767 | 169.8295 | 0.853011 | 0.28193 | 8.04E-05 | 0.002642 | RP11-162P23.2 |
| ENSG00000185215 | 291.1757 | 0.825789 | 0.224787 | 8.62E-06 | 0.000393 | TNFAIP2 |
| ENSG00000128283 | 378.2594 | 0.820312 | 0.151704 | 3.14E-09 | 3.31E-07 | CDC42EP1 |
| ENSG00000153162 | 989.0393 | 0.797564 | 0.089544 | 2.40E-20 | 9.68E-18 | BMP6 |
| ENSG00000169851 | 990.3388 | 0.79733 | 0.094256 | 1.35E-18 | 4.82E-16 | PCDH7 |
| ENSG00000125968 | 197.0893 | 0.791849 | 0.209646 | 6.54E-06 | 0.000312 | ID1 |
| ENSG00000144596 | 265.8701 | 0.78967 | 0.190624 | 1.39E-06 | 8.10E-05 | GRIP2 |
| ENSG00000163735 | 333.496 | 0.784136 | 0.163998 | 7.67E-08 | 5.86E-06 | CXCL5 |
| ENSG00000177374 | 218.6803 | 0.780477 | 0.191443 | 1.98E-06 | 0.000109 | HIC1 |
| ENSG00000077327 | 728.7609 | 0.778351 | 0.109603 | 5.82E-14 | 1.20E-11 | SPAG6 |
| ENSG00000183454 | 389.1964 | 0.777736 | 0.147405 | 6.02E-09 | 5.63E-07 | GRIN2A |
| ENSG00000188931 | 313.1346 | 0.772245 | 0.163797 | 1.08E-07 | 7.98E-06 | CFAP126 |
| ENSG00000182601 | 261.0027 | 0.768825 | 0.183111 | 1.41E-06 | 8.18E-05 | HS3ST4 |
| ENSG00000107099 | 58.79933 | 0.766787 | 0.457886 | 0.002102 | 0.036369 | DOCK8 |
| ENSG00000112379 | 1275.345 | 0.761609 | 0.079493 | 5.01E-23 | 2.78E-20 | ARFGEF3 |
| ENSG00000100678 | 400.056 | 0.761289 | 0.143868 | 5.58E-09 | 5.45E-07 | SLC8A3 |
| ENSG00000133636 | 302.1336 | 0.759908 | 0.173906 | 5.63E-07 | 3.57E-05 | NTS |
| ENSG00000107611 | 787.0891 | 0.757506 | 0.105649 | 3.65E-14 | 7.73E-12 | CUBN |
| ENSG00000115252 | 272.7472 | 0.754692 | 0.197429 | 5.28E-06 | 0.00026 | PDE1A |
| ENSG00000116016 | 168.4094 | 0.751449 | 0.236278 | 5.38E-05 | 0.001875 | EPAS1 |

| | | | | | | |
|---|---|---|---|---|---|---|
| ENSG00000187678 | 1928.609 | 0.745738 | 0.079471 | 2.90E-22 | 1.34E-19 | SPRY4 |
| ENSG00000179921 | 100.7889 | 0.743604 | 0.307967 | 0.000501 | 0.012321 | GPBAR1 |
| ENSG00000120262 | 84.37424 | 0.741924 | 0.362285 | 0.001082 | 0.021961 | CCDC170 |
| ENSG00000166016 | 682.1276 | 0.735169 | 0.110011 | 1.11E-12 | 1.97E-10 | ABTB2 |
| ENSG00000221818 | 163.7312 | 0.731601 | 0.228859 | 5.31E-05 | 0.001861 | EBF2 |
| ENSG00000164741 | 1037.052 | 0.726122 | 0.097156 | 3.85E-15 | 8.86E-13 | DLC1 |
| ENSG00000038295 | 175.9855 | 0.718925 | 0.225401 | 5.39E-05 | 0.001875 | TLL1 |
| ENSG00000198729 | 497.8926 | 0.710432 | 0.138334 | 9.40E-09 | 8.45E-07 | PPP1R14C |
| ENSG00000152760 | 465.9713 | 0.701985 | 0.132662 | 5.79E-09 | 5.49E-07 | TCTEX1D1 |
| ENSG00000142694 | 170.3532 | 0.696283 | 0.232865 | 0.000103 | 0.00328 | EVA1B |
| ENSG00000184347 | 1128.247 | 0.692125 | 0.088402 | 2.49E-16 | 7.10E-14 | SLIT3 |
| ENSG00000213085 | 265.8908 | 0.691059 | 0.192798 | 1.36E-05 | 0.00058 | CFAP45 |
| ENSG00000168421 | 518.4892 | 0.690589 | 0.141481 | 4.51E-08 | 3.60E-06 | RHOH |
| ENSG00000166596 | 147.3812 | 0.688814 | 0.257143 | 0.000248 | 0.00691 | CFAP52 |
| ENSG00000181234 | 1296.867 | 0.686537 | 0.08062 | 9.16E-19 | 3.35E-16 | TMEM132C |
| ENSG00000109625 | 137.508 | 0.686374 | 0.287842 | 0.000509 | 0.01243 | CPZ |
| ENSG00000078549 | 814.7668 | 0.685557 | 0.103598 | 1.85E-12 | 3.02E-10 | ADCYAP1R1 |
| ENSG00000138434 | 4839.154 | 0.682833 | 0.04706 | 1.01E-49 | 2.04E-46 | SSFA2 |
| ENSG00000102554 | 233.4269 | 0.679076 | 0.207927 | 4.27E-05 | 0.001543 | KLF5 |
| ENSG00000122861 | 295.373 | 0.678609 | 0.198772 | 2.45E-05 | 0.000974 | PLAU |
| ENSG00000136158 | 5060.008 | 0.6785 | 0.048125 | 1.18E-46 | 2.11E-43 | SPRY2 |
| ENSG00000091986 | 3394.952 | 0.666601 | 0.070468 | 1.69E-22 | 8.24E-20 | CCDC80 |
| ENSG00000135905 | 690.8613 | 0.6541 | 0.123725 | 5.73E-09 | 5.48E-07 | DOCK10 |
| ENSG00000118785 | 1323.134 | 0.652911 | 0.082296 | 1.22E-16 | 3.56E-14 | SPP1 |
| ENSG00000141668 | 321.0375 | 0.651119 | 0.173598 | 6.98E-06 | 0.00033 | CBLN2 |
| ENSG00000080493 | 838.1314 | 0.648905 | 0.097307 | 1.33E-12 | 2.24E-10 | SLC4A4 |
| ENSG00000198739 | 145.3181 | 0.648675 | 0.256524 | 0.000384 | 0.01003 | LRRTM3 |
| ENSG00000172985 | 132.3129 | 0.648497 | 0.280372 | 0.000623 | 0.014639 | SH3RF3 |
| ENSG00000187714 | 658.847 | 0.646699 | 0.110062 | 2.11E-10 | 2.77E-08 | SLC18A3 |
| ENSG00000167244 | 31123.93 | 0.646641 | 0.034069 | 1.28E-81 | 5.16E-78 | IGF2 |
| ENSG00000138185 | 183.4125 | 0.646149 | 0.284189 | 0.000635 | 0.014827 | ENTPD1 |
| ENSG00000099869 | 104.3963 | 0.644454 | 0.357013 | 0.001706 | 0.031198 | IGF2-AS |
| ENSG00000178695 | 5856.782 | 0.641052 | 0.048963 | 1.76E-40 | 2.58E-37 | KCTD12 |
| ENSG00000078725 | 772.8169 | 0.640304 | 0.10701 | 9.98E-11 | 1.36E-08 | BRINP1 |
| ENSG00000164056 | 3104.514 | 0.639113 | 0.053397 | 2.71E-34 | 3.64E-31 | SPRY1 |
| ENSG00000164929 | 604.5985 | 0.635712 | 0.119998 | 5.86E-09 | 5.52E-07 | BAALC |
| ENSG00000144619 | 1517.275 | 0.634665 | 0.082093 | 6.16E-16 | 1.60E-13 | CNTN4 |
| ENSG00000101222 | 184.6453 | 0.633854 | 0.219932 | 0.000147 | 0.004359 | SPEF1 |
| ENSG00000149090 | 277.0437 | 0.632522 | 0.173429 | 1.17E-05 | 0.00051 | PAMR1 |
| ENSG00000154856 | 2051.108 | 0.629559 | 0.089456 | 8.63E-14 | 1.72E-11 | APCDD1 |

| | | | | | | |
|---|---|---|---|---|---|---|
| ENSG00000154127 | 822.9059 | 0.627406 | 0.100077 | 1.94E-11 | 2.92E-09 | UBASH3B |
| ENSG00000100427 | 103.9381 | 0.627023 | 0.336121 | 0.001543 | 0.028901 | MLC1 |
| ENSG00000136286 | 133.8157 | 0.625628 | 0.278369 | 0.000753 | 0.016705 | MYO1G |
| ENSG00000159713 | 845.0526 | 0.608419 | 0.101354 | 9.46E-11 | 1.30E-08 | TPPP3 |
| ENSG00000276386 | 138.2324 | 0.606966 | 0.277394 | 0.000865 | 0.018608 | CNTNAP3P2 |
| ENSG00000106714 | 1594.954 | 0.604632 | 0.074296 | 1.97E-17 | 6.09E-15 | CNTNAP3 |
| ENSG00000244405 | 2535.474 | 0.603236 | 0.060006 | 5.90E-25 | 4.32E-22 | ETV5 |
| ENSG00000004838 | 384.1246 | 0.589109 | 0.148293 | 3.26E-06 | 0.000169 | ZMYND10 |
| ENSG00000176601 | 145.6949 | 0.587654 | 0.276567 | 0.001079 | 0.021961 | MAP3K19 |
| ENSG00000167711 | 143.1948 | 0.585099 | 0.28729 | 0.001195 | 0.023807 | SERPINF2 |
| ENSG00000154080 | 113.8952 | -0.58588 | 0.34417 | 0.002128 | 0.03662 | CHST9 |
| ENSG00000196277 | 90.00304 | -0.59387 | 0.368341 | 0.002436 | 0.040873 | GRM7 |
| ENSG00000163637 | 492.8375 | -0.59492 | 0.150527 | 3.58E-06 | 0.000183 | PRICKLE2 |
| ENSG00000137501 | 313.4755 | -0.5952 | 0.184637 | 4.98E-05 | 0.001755 | SYTL2 |
| ENSG00000197747 | 872.4758 | -0.60391 | 0.0974 | 3.31E-11 | 4.89E-09 | S100A10 |
| ENSG00000132849 | 242.9018 | -0.60985 | 0.196424 | 6.42E-05 | 0.002191 | PATJ |
| ENSG00000172572 | 1989.736 | -0.61453 | 0.067589 | 5.10E-21 | 2.16E-18 | PDE3A |
| ENSG00000075223 | 978.4148 | -0.62114 | 0.108048 | 4.70E-10 | 5.74E-08 | SEMA3C |
| ENSG00000129151 | 233.6159 | -0.62154 | 0.192866 | 5.10E-05 | 0.00179 | BBOX1 |
| ENSG00000147437 | 83.83388 | -0.63459 | 0.379168 | 0.002116 | 0.036577 | GNRH1 |
| ENSG00000145721 | 655.9064 | -0.64058 | 0.119943 | 3.52E-09 | 3.64E-07 | LIX1 |
| ENSG00000165376 | 396.3021 | -0.65838 | 0.148707 | 4.22E-07 | 2.77E-05 | CLDN2 |
| ENSG00000021645 | 403.4302 | -0.69222 | 0.143164 | 6.09E-08 | 4.72E-06 | NRXN3 |
| ENSG00000143858 | 178.7474 | -0.69381 | 0.245054 | 0.000162 | 0.004757 | SYT2 |
| ENSG00000152689 | 213.7669 | -0.70994 | 0.246174 | 0.000142 | 0.004256 | RASGRP3 |
| ENSG00000124749 | 1052.756 | -0.714 | 0.095377 | 3.50E-15 | 8.17E-13 | COL21A1 |
| ENSG00000156535 | 226.431 | -0.71657 | 0.195722 | 9.58E-06 | 0.000431 | CD109 |
| ENSG00000166106 | 1867.276 | -0.71805 | 0.077178 | 6.65E-22 | 2.97E-19 | ADAMTS15 |
| ENSG00000115414 | 33626.06 | -0.72172 | 0.03148 | 1.30E-117 | 1.04E-113 | FN1 |
| ENSG00000169436 | 1037.296 | -0.73462 | 0.095281 | 6.15E-16 | 1.60E-13 | COL22A1 |
| ENSG00000154027 | 495.1722 | -0.73836 | 0.15502 | 7.68E-08 | 5.86E-06 | AK5 |
| ENSG00000145708 | 134.7128 | -0.73989 | 0.331366 | 0.000787 | 0.017284 | CRHBP |
| ENSG00000143248 | 1905.923 | -0.76864 | 0.090187 | 5.98E-19 | 2.29E-16 | RGS5 |
| ENSG00000115232 | 575.0555 | -0.77941 | 0.138689 | 8.39E-10 | 9.73E-08 | ITGA4 |
| ENSG00000125378 | 234.8834 | -0.79411 | 0.19975 | 2.92E-06 | 0.000153 | BMP4 |
| ENSG00000177459 | 146.2749 | -0.79738 | 0.250426 | 5.39E-05 | 0.001875 | ERICH5 |
| ENSG00000143768 | 113.5148 | -0.8035 | 0.297635 | 0.000226 | 0.006378 | LEFTY2 |
| ENSG00000187098 | 217.0607 | -0.80761 | 0.214099 | 6.38E-06 | 0.000306 | MITF |
| ENSG00000144285 | 137.9672 | -0.8492 | 0.28998 | 0.000115 | 0.003544 | SCN1A |

| | | | | | | |
|---|---|---|---|---|---|---|
| ENSG00000108821 | 16084.3 | -0.86553 | 0.040781 | 1.36E-100 | 7.29E-97 | COL1A1 |
| ENSG00000078098 | 87.90229 | -0.91471 | 0.332667 | 0.000189 | 0.005455 | FAP |
| ENSG00000113805 | 331.8 | -0.93774 | 0.174569 | 3.28E-09 | 3.43E-07 | CNTN3 |
| ENSG00000168743 | 50.91675 | -0.98879 | 0.478676 | 0.000969 | 0.020238 | NPNT |
| ENSG00000183145 | 451.5226 | -1.01527 | 0.136948 | 5.67E-15 | 1.27E-12 | RIPPLY3 |
| ENSG00000178568 | 93.40963 | -1.06706 | 0.388354 | 0.000185 | 0.00534 | ERBB4 |
| ENSG00000047457 | 672.7094 | -1.07489 | 0.113198 | 9.42E-23 | 4.74E-20 | CP |
| ENSG00000167306 | 360.9299 | -1.17427 | 0.156212 | 2.30E-15 | 5.60E-13 | MYO5B |
| ENSG00000245112 | 108.8179 | -1.2822 | 0.318217 | 1.75E-06 | 9.81E-05 | SMARCA5-AS1 |
| ENSG00000249307 | 45.27171 | -1.38792 | 0.503317 | 0.000182 | 0.005277 | LINC01088 |
| ENSG00000213886 | 98.94692 | -1.44704 | 0.355736 | 1.98E-06 | 0.000109 | UBD |
| ENSG00000204531 | 108.2005 | -1.46529 | 0.298986 | 3.54E-08 | 2.91E-06 | POU5F1 |
| ENSG00000184697 | 88.82106 | -1.53365 | 0.329485 | 1.24E-07 | 9.00E-06 | CLDN6 |
| ENSG00000041353 | 128.6722 | -1.54224 | 0.278986 | 1.35E-09 | 1.51E-07 | RAB27B |
| ENSG00000167600 | 25.60487 | -1.61805 | 0.672568 | 0.0004 | 0.01039 | CYP2S1 |
| ENSG00000102755 | 53.57856 | -1.75994 | 0.431463 | 1.51E-06 | 8.63E-05 | FLT1 |
| ENSG00000112782 | 35.13461 | -1.85181 | 0.551054 | 2.97E-05 | 0.001144 | CLIC5 |
| ENSG00000265992 | 119.8317 | -1.88208 | 0.268741 | 1.08E-13 | 2.12E-11 | ESRG |
| ENSG00000198796 | 54.03718 | -2.16171 | 0.462274 | 1.11E-07 | 8.14E-06 | ALPK2 |
| ENSG00000241186 | 49.46296 | -2.28873 | 0.464355 | 3.29E-08 | 2.72E-06 | TDGF1 |
