# Supplementary material for: A Novel Vitronectin Peptide Facilitates Differentiation of Oligodendrocytes from Human Pluripotent Stem Cells (Synthetic ECM for Oligodendrocyte Differentiation)
Source: Biology (Basel). 2021 Dec 1;10(12):1254. doi: 10.3390/biology10121254 (PMC8698880; doi:10.3390/biology10121254)
Supplement: Supplementary file 1 [file biology-10-01254-s001.zip › Supplementary Figures_Park et al_R1.pptx]

## Slide 1
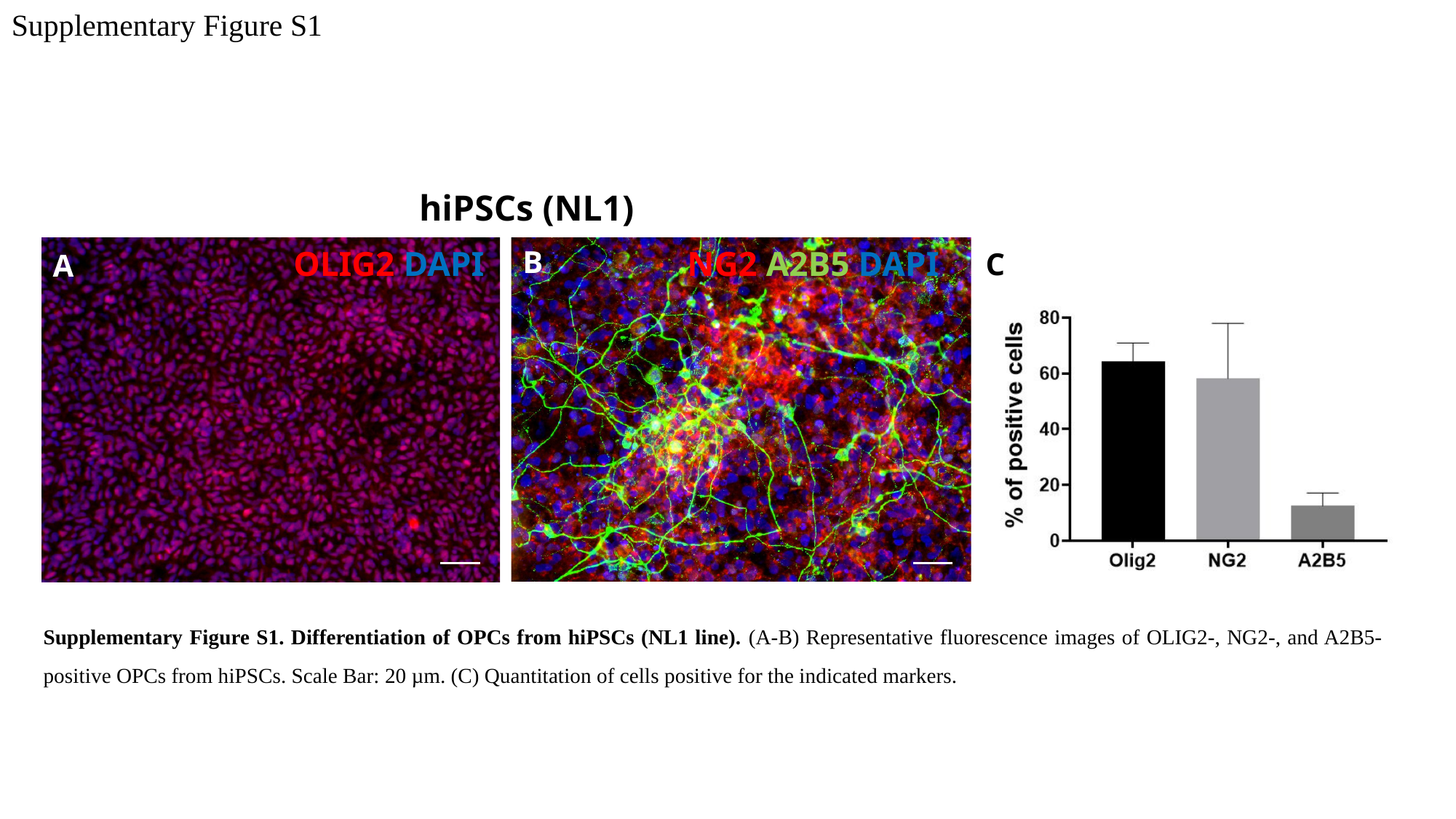

Supplementary Figure S1
hiPSCs (NL1)
B
OLIG2 DAPI
NG2 A2B5 DAPI
C
A
Supplementary Figure S1. Differentiation of OPCs from hiPSCs (NL1 line). (A-B) Representative fluorescence images of OLIG2-, NG2-, and A2B5-positive OPCs from hiPSCs. Scale Bar: 20 µm. (C) Quantitation of cells positive for the indicated markers.

## Slide 2
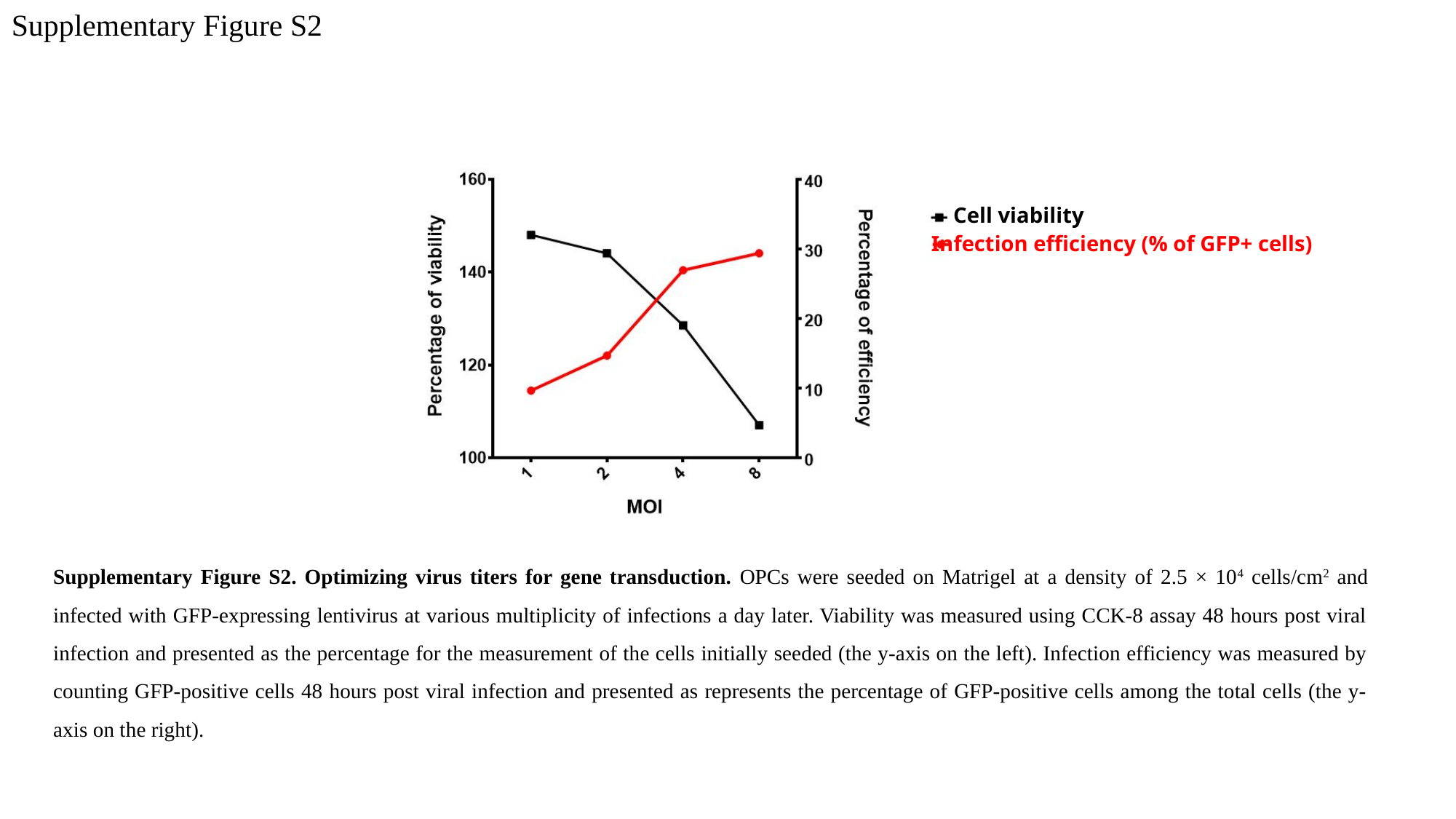

Supplementary Figure S2
Cell viability
Infection efficiency (% of GFP+ cells)
Supplementary Figure S2. Optimizing virus titers for gene transduction. OPCs were seeded on Matrigel at a density of 2.5 × 10^4^ cells/cm2 and infected with GFP-expressing lentivirus at various multiplicity of infections a day later. Viability was measured using CCK-8 assay 48 hours post viral infection and presented as the percentage for the measurement of the cells initially seeded (the y-axis on the left). Infection efficiency was measured by counting GFP-positive cells 48 hours post viral infection and presented as represents the percentage of GFP-positive cells among the total cells (the y-axis on the right).

## Slide 3
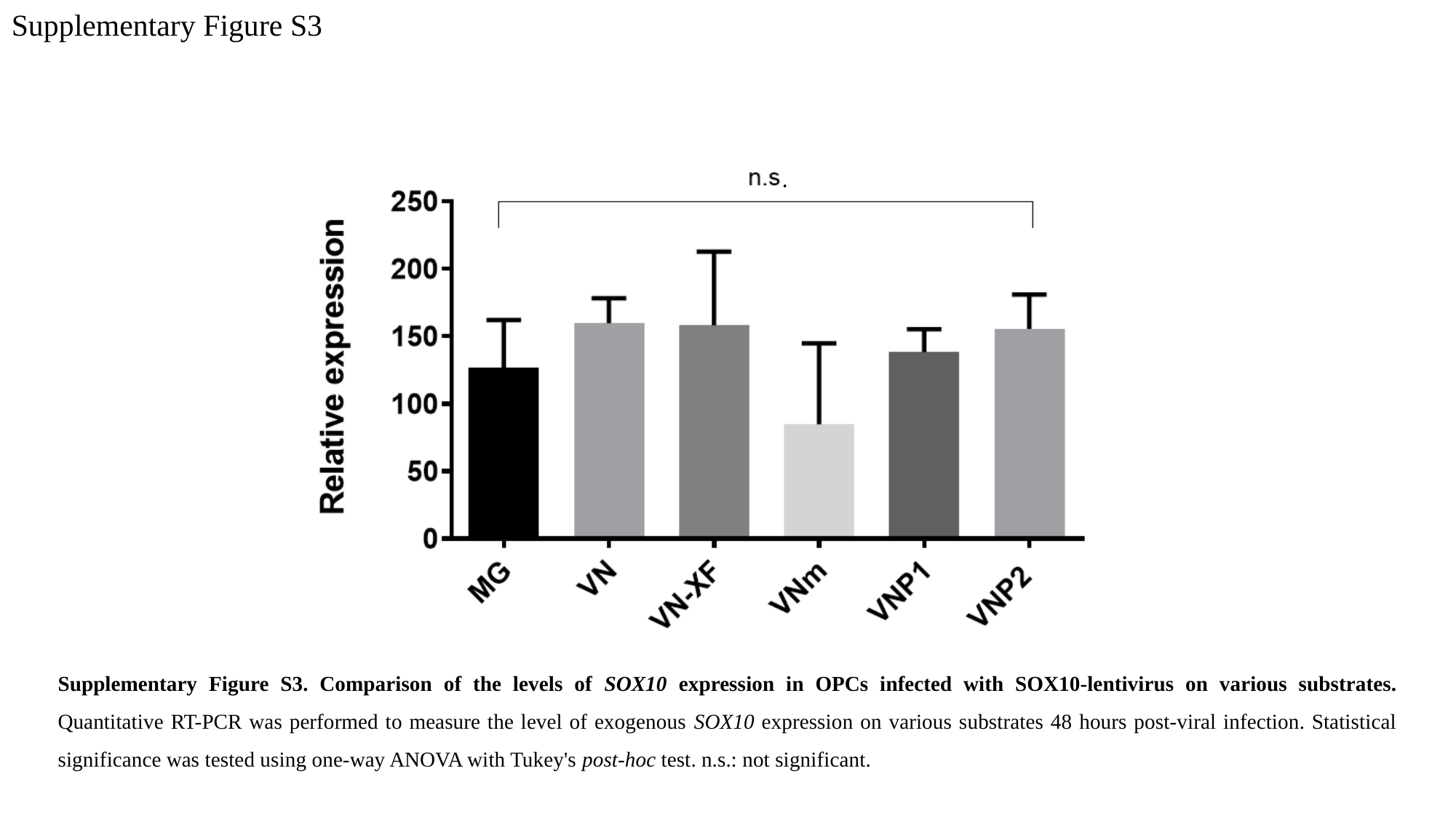

Supplementary Figure S3
.
Supplementary Figure S3. Comparison of the levels of SOX10 expression in OPCs infected with SOX10-lentivirus on various substrates. Quantitative RT-PCR was performed to measure the level of exogenous SOX10 expression on various substrates 48 hours post-viral infection. Statistical significance was tested using one-way ANOVA with Tukey's post-hoc test. n.s.: not significant.

## Slide 4
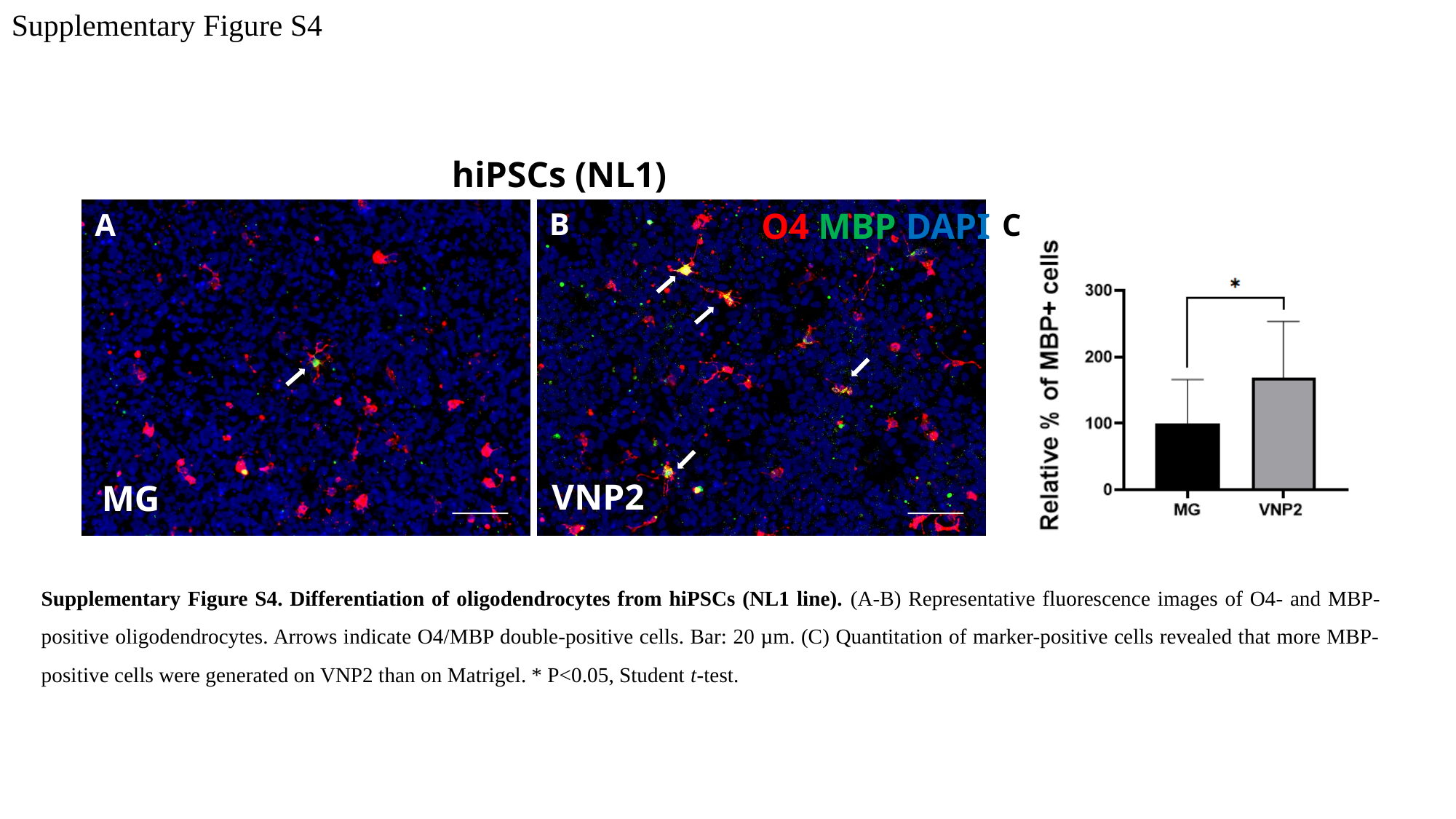

Supplementary Figure S4
hiPSCs (NL1)
O4 MBP DAPI
B
A
C
VNP2
MG
Supplementary Figure S4. Differentiation of oligodendrocytes from hiPSCs (NL1 line). (A-B) Representative fluorescence images of O4- and MBP-positive oligodendrocytes. Arrows indicate O4/MBP double-positive cells. Bar: 20 µm. (C) Quantitation of marker-positive cells revealed that more MBP-positive cells were generated on VNP2 than on Matrigel. * P<0.05, Student t-test.

## Slide 5
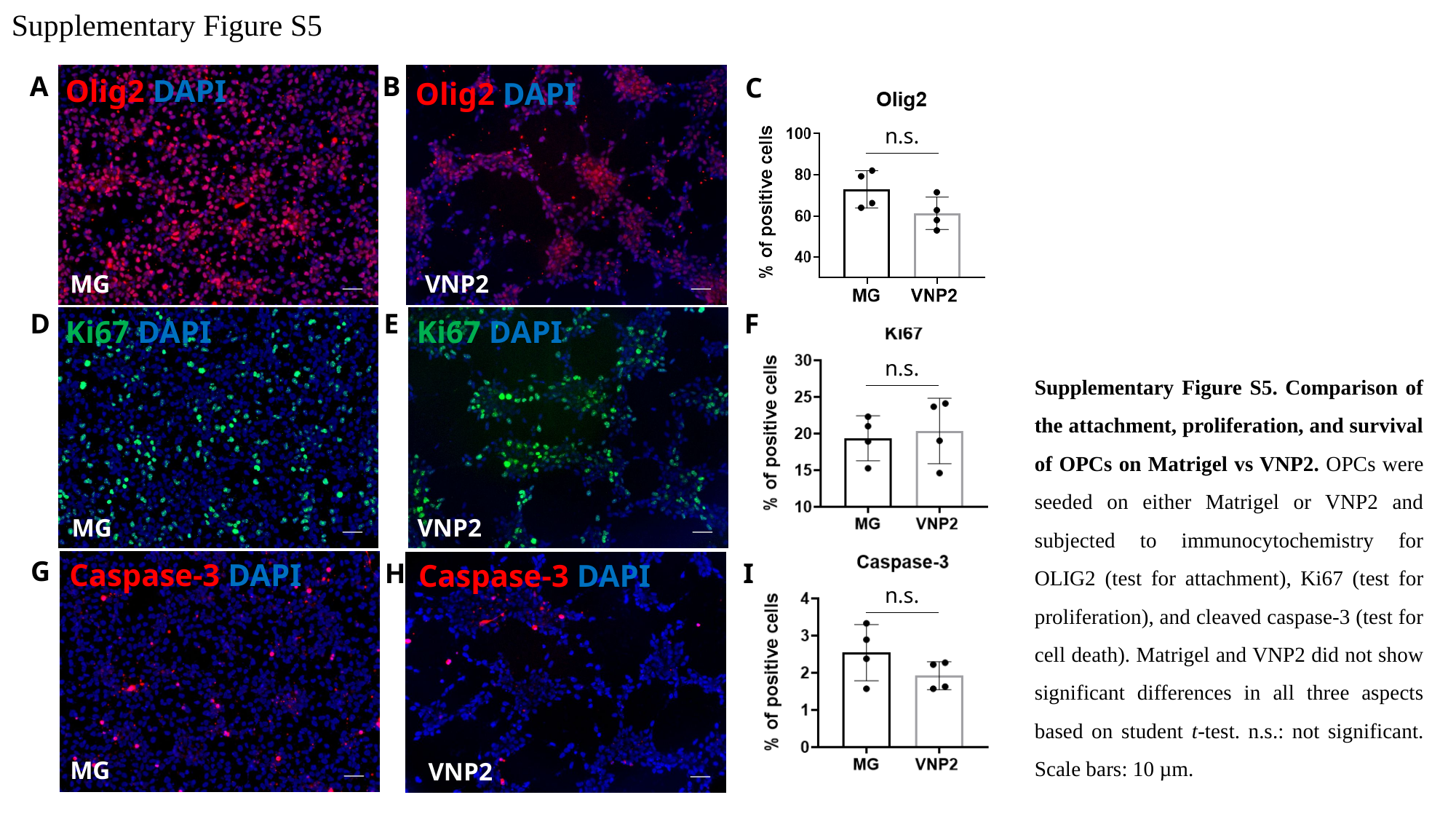

Supplementary Figure S5
B
A
C
Olig2 DAPI
Olig2 DAPI
n.s.
MG
VNP2
D
E
F
Ki67 DAPI
Ki67 DAPI
n.s.
Supplementary Figure S5. Comparison of the attachment, proliferation, and survival of OPCs on Matrigel vs VNP2. OPCs were seeded on either Matrigel or VNP2 and subjected to immunocytochemistry for OLIG2 (test for attachment), Ki67 (test for proliferation), and cleaved caspase-3 (test for cell death). Matrigel and VNP2 did not show significant differences in all three aspects based on student t-test. n.s.: not significant. Scale bars: 10 µm.
MG
VNP2
G
Caspase-3 DAPI
H
I
Caspase-3 DAPI
n.s.
MG
VNP2

## Slide 6
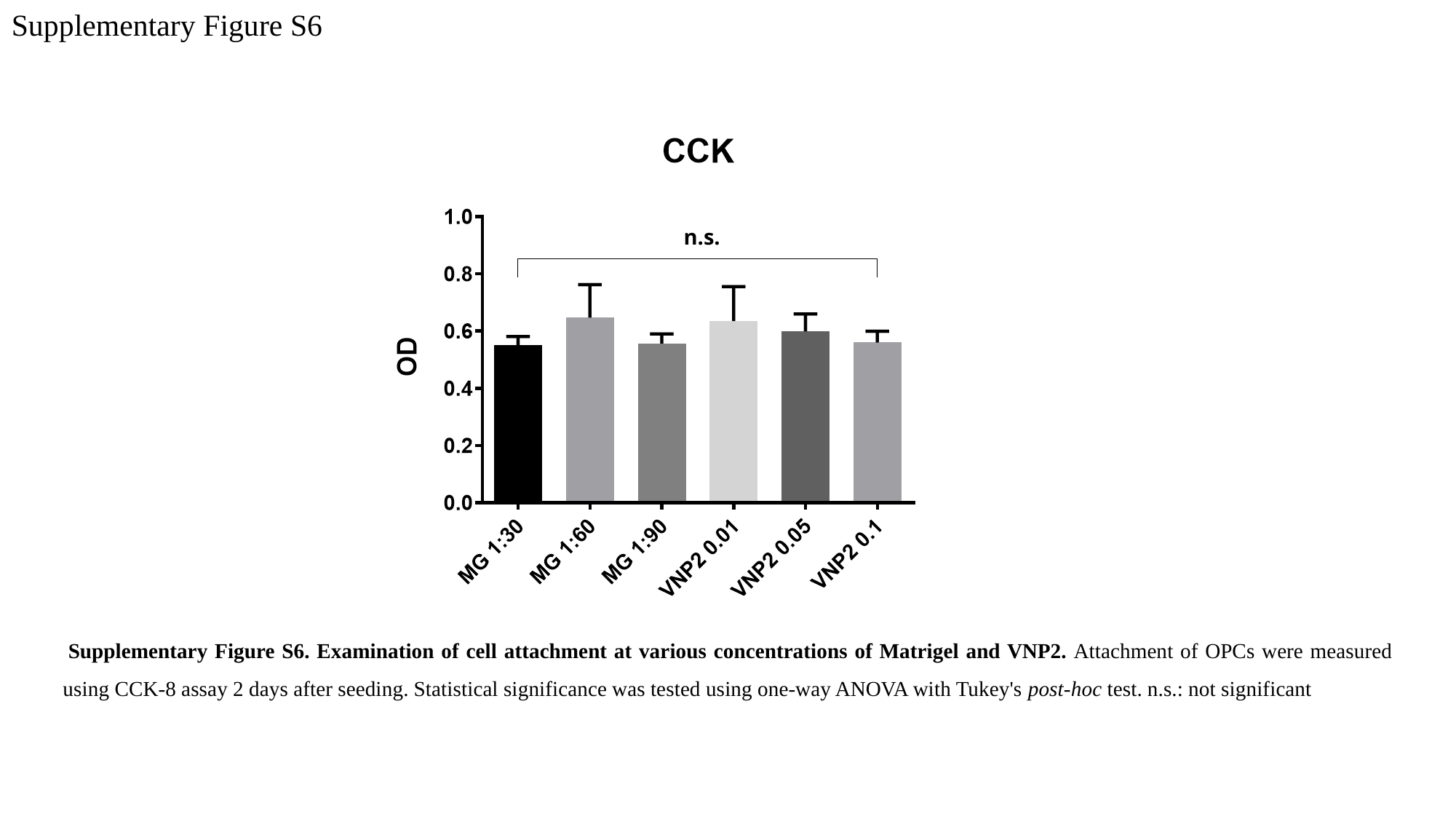

Supplementary Figure S6
n.s.
 Supplementary Figure S6. Examination of cell attachment at various concentrations of Matrigel and VNP2. Attachment of OPCs were measured using CCK-8 assay 2 days after seeding. Statistical significance was tested using one-way ANOVA with Tukey's post-hoc test. n.s.: not significant

## Slide 7
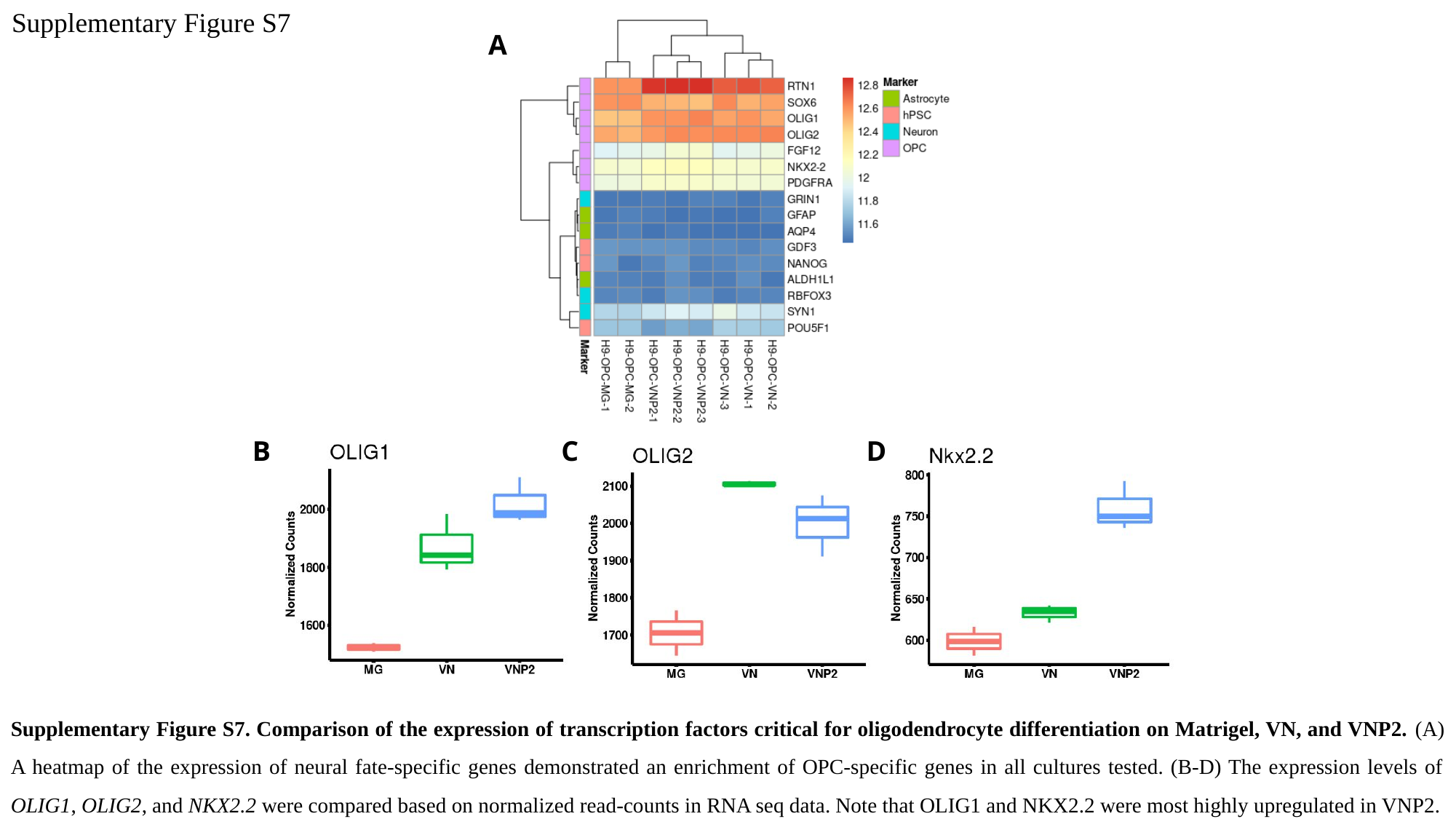

Supplementary Figure S7
A
B
C
D
Supplementary Figure S7. Comparison of the expression of transcription factors critical for oligodendrocyte differentiation on Matrigel, VN, and VNP2. (A) A heatmap of the expression of neural fate-specific genes demonstrated an enrichment of OPC-specific genes in all cultures tested. (B-D) The expression levels of OLIG1, OLIG2, and NKX2.2 were compared based on normalized read-counts in RNA seq data. Note that OLIG1 and NKX2.2 were most highly upregulated in VNP2.

## Slide 8
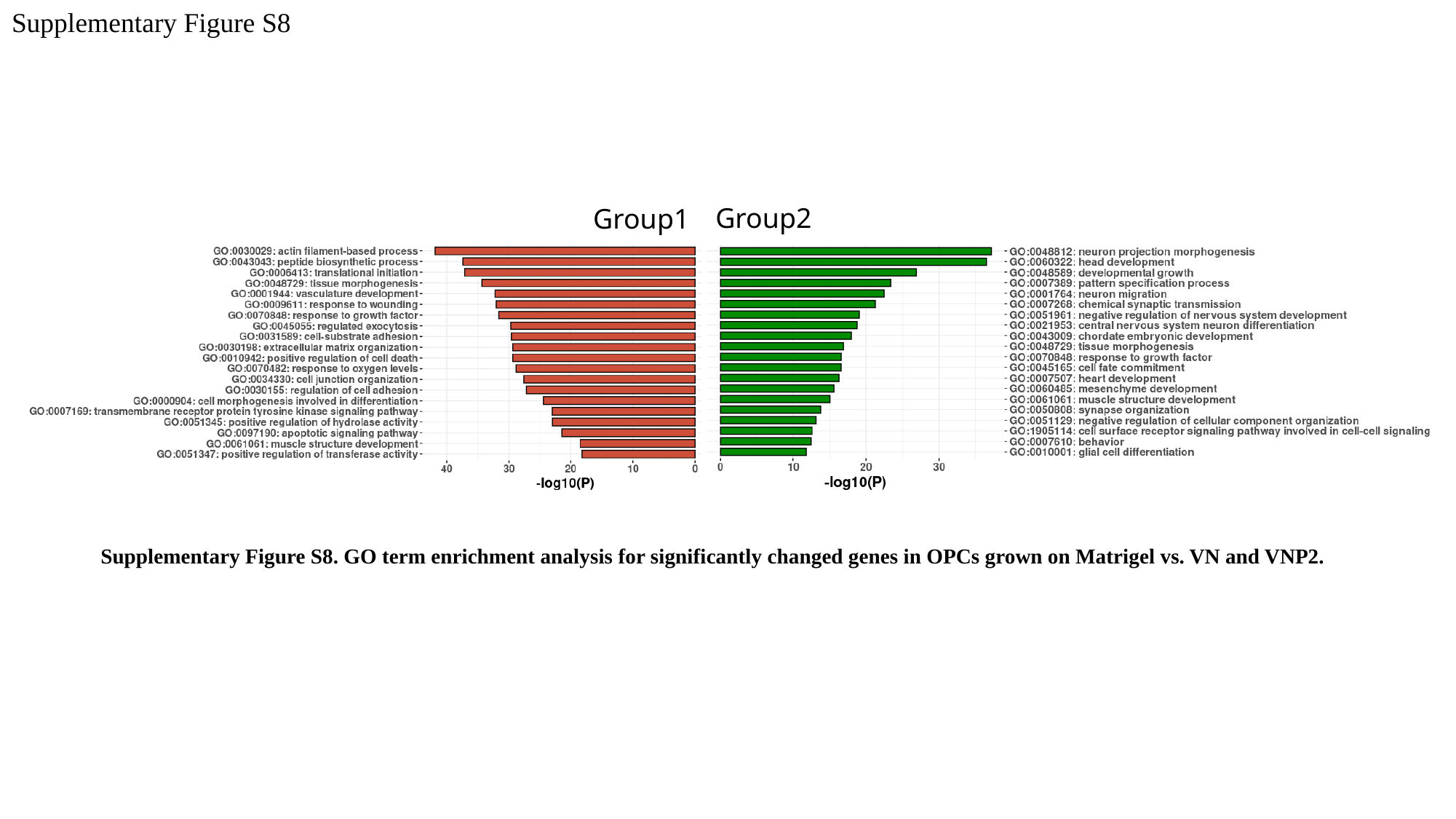

Supplementary Figure S8
Group2
Group1
Supplementary Figure S8. GO term enrichment analysis for significantly changed genes in OPCs grown on Matrigel vs. VN and VNP2.
